# Family life is critical for migration and survival in a long-lived social bird

**DOI:** 10.1007/s00265-026-03787-5

**Published:** 2026-08-20

**Authors:** Hans Linssen, E. Emiel van Loon, Robert E. Wilson, Sarah A. Sonsthagen, Götz Eichhorn, Judy Z. Shamoun-Baranes, Bart A. Nolet

**Affiliations:** 1 Department of Theoretical and Computational Ecology, Institute for Biodiversity and Ecosystem Dynamics, University of Amsterdam, Amsterdam, Noord-Holland, The Netherlands; 2 Department of Animal Ecology, Netherlands Institute of Ecology, Wageningen, Gelderland, The Netherlands; 3 Nebraska State Museum and School of Natural Resources, University of Nebraska-Lincoln, Lincoln, NE USA; 4 U.S. Geological Survey, Nebraska Cooperative Fish and Wildlife Research Unit, School of Natural Resources, University of Nebraska-Lincoln, Lincoln, NE USA; 5 Michael-Otto-Institut im NABU, Bergenhusen, Schleswig-Holstein, Germany

**Keywords:** Bewick’s swan, Disturbance, Ontogeny, Parent-offspring associations, Social learning, Waterfowl

## Abstract

Extended parental care is common among birds and particularly well known among long-lived migratory species. Yet, factors influencing the duration of parental care are poorly understood, and empirical evidence for the functional importance of family life for offspring development is scarce. Using unique tracking and genetic data of 55 adult and juvenile migratory Bewick’s swans, among which 18 parent-offspring pairs and 13 sibling pairs, we quantify variation in family bond duration and identify the cause and consequences of their dissolution. Families generally broke up during the second half of the first northward migration of the offspring, when offspring were nearly one year old. Earlier separation occurred on the wintering grounds in response to disturbance (*n* = 10 juveniles) and led to delayed migration and, critically, lower survival (40 vs. 86%). Our results provide rare empirical evidence that family bonds, as a pathway for social learning, are not just important, but vital to the migration and survival of young family-living birds.

**Supplementary Information:**

The online version contains supplementary material available at https://doi.org/10.1007/s00265-026-03787-5.

## Significance statement

Although many animal species continue to stay in family groups after the young become independent, evidence for the functional importance of such extended parental care to offspring development remains limited. Using GPS tracking and genetic data, we show that juvenile Bewick’s swans rely on continued family associations for successful migration and higher chances of survival throughout their first year. Our findings provide rare empirical support for the critical role of extended parental care and identify family life as a key pathway for social learning. Furthermore, we underscore the need to minimise disturbance of family-living animals during this sensitive life stage.

**Supplementary Information:**

The online version contains supplementary material available at https://doi.org/10.1007/s00265-026-03787-5.

## Introduction

Social learning is widespread across the animal kingdom, with behaviours of young individuals taking shape through interactions with kin and non-kin in their social environment (Heyes 1994; Galef and Laland 2005; Aplin 2019; Aikens et al. 2022; Newton 2024). One primary form of social learning occurs through extended parental care, where offspring continue to associate with and receive guidance from their parents after leaving the natal site. Among birds, this phenomenon is best known in long-lived migratory species such as waterfowl (Kear 1970; Owen 1977; Scott 1980; Warren et al. 1993; Kölzsch et al. 2020; Wilson et al. 2025), cranes (Johnsgard 1983; Mueller et al. 2013) and terns (Ashmole and Tovar 1968; Feare 1975; Byholm et al. 2022), where parents can help their offspring learn migration routes (Byholm et al. 2022) and support them during long-distance movements by leading flight formations (Kölzsch et al. 2020). Still, there is little empirical evidence for the functional importance of extended parental care to the migration success and survival of young family-living birds (Weegman et al. 2016; see Byholm et al. 2022). As a result, the importance of extended parental care as a pathway for social learning remains poorly understood.

Patterns of extended parental care can vary substantially within species. For example, in Svalbard-breeding barnacle geese (*Branta leucopsis*), about three-quarters of juveniles were still associated with their parents at the onset of their first northward migration from Britain (Black and Owen 1989), whereas families in the neighbouring Baltic-Russian flyway population dissolved on the wintering grounds along the Wadden Sea well before departure, with virtually no families migrating north together (Jonker et al. 2011). Furthermore, greater white-fronted geese (*Anser albifrons*) are known to have family bonds lasting up to 13 years (Warren et al. 1993; Weegman et al. 2016), and 70% of second-winter and 40% of third- and fourth-winter birds still resided with their parents in a population wintering in North America (Ely 1993). In contrast, the subspecies wintering in Europe showed much earlier family break-ups during the juveniles’ first winter, which were presumably triggered by disturbance events (Gupte et al. 2019). Clearly, diversity exists in the duration of parent-offspring associations among migratory birds, but the causes and consequences of this diversity remain poorly understood.

We use unique tracking data of parent-offspring and sibling pairs from families of Bewick’s swan (*Cygnus columbianus bewickii*) to examine the spatiotemporal patterns of family break-ups and subsequent reunions, and effects of social learning through extended parental care on juvenile migration and survival. Bewick’s swans are long-lived, Arctic-breeding migratory birds known for their family associations (Scott 1980; Earnst and Bart 1991; Rees 2006a). Juveniles migrate south with their parents and are generally thought to remain with them throughout the non-breeding season and part of their first northward migration (Rees 2006b). Parent-offspring and sibling pairs are also known to sometimes reunite at the wintering grounds in later winters (Rees 2006a). Such observations support the widespread notion that family life is important for the offspring, parents, or both. However, they are limited to families or parent-offspring pairs that can be visually identified as such at specific field sites (i.e., observed in close proximity), and often miss cases of temporary or permanent separation.

We infer family relationships on the basis of genetic data, independent from tracks or field observations. Several juveniles were inadvertently separated from their families prematurely during winter because of disturbance events, introducing a quasi-experimental element to our study. We compare the first northward migration (return to the Arctic breeding grounds) and survival of these early-separated juveniles (“orphans”) to those that naturally maintained longer associations with their parents. By integrating patterns of family cohesion with data on migration and survival, we provide novel insights into the importance of family life as a pathway for social learning and development in long-lived migrants.

## Methods

### Study area and tagging

The Western Palearctic flyway population of Bewick’s swans breeds on the tundra along the Pechora Sea coast in European Russia and winters in the countries along the North Sea (Beekman et al. 2019; Rees et al. 2019). Its winter distribution has been shifting north-eastwards under the influence of milder winters, and the centre is currently located in northern Germany (Nuijten et al. 2020b; Linssen et al. 2023, 2024; Koffijberg et al. 2025). Juveniles travel with their parents to the wintering grounds, where they generally remain as family groups within larger flocks (Scott 1980; Rees 2006a).

We captured Bewick’s swans with cannon nets while flock-feeding on agricultural land in the Netherlands in the winters of 2016/17–2022/23, and in Germany (Lower Saxony) in the winters of 2021/22 and 2022/23. We conducted 14 catches in total, with 16.5 ± 10.7 (mean ± SD) individuals caught per catch. During those catches, we fitted 148 Bewick’s swans in total with solar-powered GPS-GSM transmitter neck collars. Before 2020/21 mostly adult females and juvenile males were tagged (sex determined on site by inspection of the cloaca); from that winter onwards, juvenile females and some adult males were also tagged with the goal of tracking families. The neck collars weighed 70 g (2016/17-2019/20; MadebyTheo, custom-made) or 65 g (2020/21–2022/23; Ornitela, OrniTrack-N55), amounting to 0.8%–1.6% of the body mass of the adult swans and 1.0%–1.6% of that of the juveniles. Blood samples for DNA analysis were collected from the medial metatarsal vein or the brachial vein. After handling, all birds from a catch were released together at nearby known night roost sites. The transmitters collected GPS locations with a frequency between 2 h and 5 min (accuracy on average 3.6 m; Nuijten et al. 2014), depending on battery charge, and sent their data to servers through the GSM network. Due to the absence of GSM network coverage in the breeding range, any GPS data recorded during summer are transferred in autumn upon returning into the GSM network.

### Genetics library preparation and bioinformatics

We used the blood samples to identify familial relationships among the tagged birds. Genomic DNA was extracted using a FavorPrep tissue genomic DNA extraction kit (Favorgen). Extractions were quantified using a NanoDrop One spectrophotometer (Thermo Scientific). We followed DaCosta and Sorenson (2014) for double digest restriction-site associated DNA (ddRAD) library preparation and bioinformatic pipelines (Python scripts available at http://github.com/BU-RAD-seq/ddRAD-seq-Pipeline, accessed on 22 May 2024) with single-end sequencing (150 bp) completed on a NovaSeq X Plus (Admera Health, South Plainfield, NJ, USA). The bioinformatic steps we performed are outlined in detail in DaCosta and Sorenson (2014). Briefly, after quality control filtering and clustering of similar reads into putative loci, we used blastn v.2 (Altschul et al. 1990) to determine the genomic positions of clusters relative to the mute swan (*Cygnus olor*) reference genome (GenBank assembly GCA_009769625.2; bCygOlo1.pri.v2). After the alignment step, we used a Python script (RADGenotypes.py) to genotype individuals at each locus. Individuals were scored as heterozygous if two distinct haplotypes (i.e., alleles) each accounted for > 29% of sequence reads for a given sample, or if the second allele was represented by 20–29% of reads and present in other individuals. Loci across all samples with a median per sample sequencing depth ≥ 10, >95% good genotype, < 5% missing genotypes, and < 5% flagged genotypes were retained for downstream analyses. Raw Illumina reads are accessioned on the National Center for Biotechnology Information (NCBI) Sequence Read Archive (BioProject: PRJNA1290665, Biosample accessions: SAMN49931523-SAMN49931756).

We confirmed the cloacal sexing of birds in the field by using the presence of loci located on W chromosome from the ddRAD dataset. In birds, females are the heterogametic sex (ZW) and therefore sequence reads of putative W loci should show the pattern of greater than 0 in females and 0 or nearly so in males. The two loci that had blast analysis results for the W chromosome of mute swans (Genbank accession: NC_049199.1) had a median number of reads of 27 and 261 and an average of 29.9 and 236.8 for females, and median 0 and average < 0.5 reads for males.

### Identifying familial relationships

We expected high familial relatedness in the dataset and therefore employed the clustering algorithm in the program fineRADstructure (Malinsky et al. 2018) with autosomal loci, which clusters individuals based on the sharing of identical or nearest-neighbour haplotypes at each locus using a recent co-ancestry approach. We expected individuals that are genetically related to share a higher co-ancestry value and form distinct clusters (i.e., family groupings) within the population. We used all single nucleotide polymorphisms (SNPs) within each locus for the analysis. SNPs were concatenated to define haploblocks, and the proportion of missing alleles per individual ranged from 0.000 to 0.023 (median 0.002 per individual). Samples were assigned to populations using 1,000,000 iterations sampled every 1,000 steps with a burn-in of 500,000. We used 1,000,000 iterations of the tree-building algorithm to define and assess the relationships among clusters. The output was visualized using the R scripts fineradstructureplot.r and finestructurelibrary.r (http://github.com/millanek/fineRADstructure, accessed on 27 May 2024).

We used the program COLONY v.2.0.6.8 (Wang and Santure 2009; Jones and Wang 2010) to assign parentage and siblingship. Our data set was likely to comprise multi-year familial associations, so all individuals irrespective of age were included in the pool of candidate parents to aid in the identification of multi-generational groups. Starting priors for allelic dropout and other genotype errors did not influence familial relationship estimates due to the large sample of loci in the ddRAD data. We performed the analysis using allelic data with a monogamous mating system as well as polygamy to allow for half-sibling relationships.

### Identifying family break-ups and reunions

**Fig. 1:**
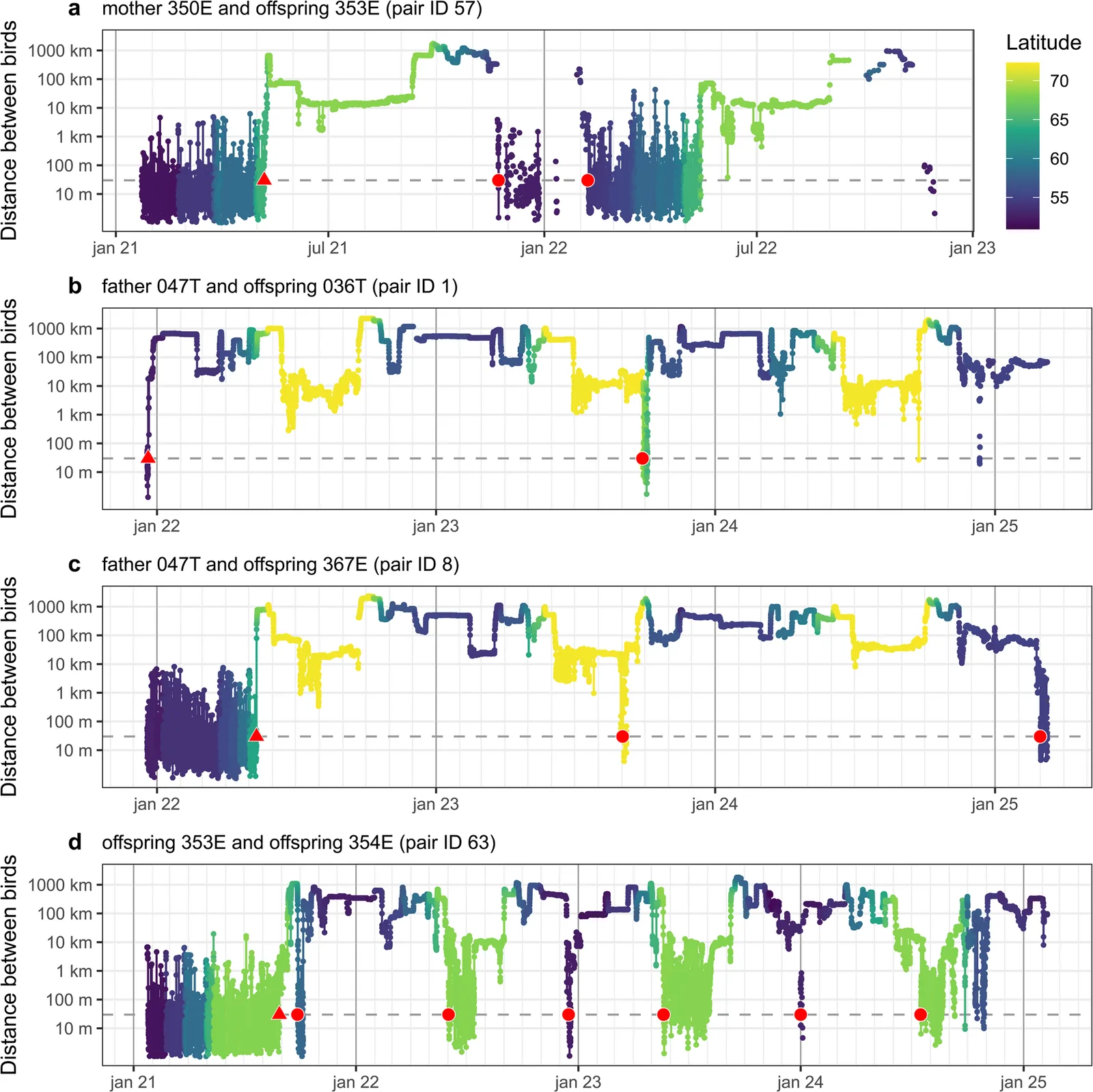
Examples of the distance between **a-c)** parent-offspring pairs and **d)** one sibling pair over the years, revealing initial break-ups (red triangles) and subsequent reunions (beginnings marked with red circles) in related Bewick’s swans. Each time series starts at the date of capture, which is in the offspring’s first winter season. Colours denote the latitude of the first bird of the pair; the maps in Fig. 2 provide a visual reference for latitude. The grey dashed line at 30 m indicates the threshold used for assessing break-ups and reunions. **(a)** Daughter 353E spends her second winter (2021/22) with her mother 350E and travels with her to the breeding range for her full second northward migration (2022). **(b)** Daughter 036T gets separated from her family when her mother (027T) is killed on the wintering roost in December 2021. She encounters her father (047T) almost two years later (September 2023) on Novaya Zemlya and they travel south together for one week. **(c)** In the same family as **b**, another daughter (367E) remains with her father (047T) after the disturbance. They also reunite in the summer of 2023, but separate before travelling south. **(d)** Siblings 353E and 354E reunite every summer in the Pechora Bay and also shortly during their third and fourth winter in the Netherlands. They even travel the first part of their fifth autumn migration (2024) together. Plots of hourly distances for all related bird pairs are provided in the Supplementary Information 2. Fig. created using RStudio (R Core Team 2023)

For each GPS-tracked bird, we calculated the median hourly coordinates along its entire track. We then calculated hourly distances between related bird pairs (either parent-offspring or siblings; Fig. 1). We defined the break-up date for each pair as the day before the first day on which their distance consistently exceeded 30 m, a threshold selected because it well exceeds the spatial accuracy of our GPS transmitters while still representing ecologically meaningful proximity as observed in the field. The exact time and location of break-up were derived by taking the last hourly position where the birds were still within 30 m from each other. In two cases, a parent and offspring got temporarily separated due to a roost site disturbance and later rejoined on the wintering ground (see Methods section Inadvertent family break-ups). In these cases, we ignored the initial period of separation, instead identifying the second, definitive separation as the true break-up moment.

To identify reunions (sustained periods of proximity indicating active social interaction rather than chance co-location within the same flock), we searched for intervals after break-up during which birds were predominantly within 30 m of each other (Supplementary Information 1 Fig. S1). If such proximity extended for more than two days, we considered the likelihood of chance co-location to be negligible and classified the event as a reunion. If it lasted two days or less, i.e., when the likelihood of misclassifying chance proximity as a reunion was higher, we visually examined the GPS tracks to assess whether the birds followed one another (in which case we classified it as a reunion). All instances of very brief proximity (up to a few hours) showed no clear coordinated movement and were thus not classified as reunions. The relatively high spatial accuracy of our GPS transmitters ensured that our reunion assessments were robust to measurement error. Plots of hourly distances for all related bird pairs are provided in the Supplementary Information 2.

### Inadvertent family break-ups

On 18 December 2021, two days after a successful catching and tagging event, hunters entered the nature reserve that contained the night roost where we had released the tagged swans. Six tagged individuals were killed (possibly along with an unknown number of unmarked birds or marked birds without GPS transmitters), causing an extreme disturbance to the swans present on or near the roost, which was also apparent in our tracking data. The GPS transmitters recorded one family where the mother (027T) was killed and one offspring (036T) got separated from her father (047T) and siblings (Fig. 1b), meaning that 036T prematurely became orphaned.

That same winter of 2021/22, a total of nine other tagged juveniles were observed in the field to be without parents, despite having resided in flocks of Bewick’s swans at their moment of catching (Supplementary Information 1 Table S1). Five of these juveniles belonged to the catching event that was quickly followed by the hunting disturbance; likely their parents were killed or they separated during or in the aftermath of the event. The other four belonged to later catches during the same winter in another area. The genetic analysis indicated that we had not caught any parent of these four juveniles, suggesting that the catching event split them from their family. Moreover, none of the parents of these nine orphaned juveniles had been tagged, so we could not determine break-ups using our method described above, nor identify possible reunions in the following years. We nevertheless included them in our break-up analysis, assuming that the moment and location of their break-up are those of their respective catching events. We observed the juveniles in the field to establish whether or not they associated with other swans while in the wintering range. For an overview of all ten orphaned juveniles, see Supplementary Information 1 Table S1.

We identified no orphaned juveniles other than the ten GPS-tagged ones. However, since our efforts of observing family status were restricted primarily to GPS-tagged individuals, we do not know how often loss of parents occurs in the wider population although it is probably rare (Rees 2006b).

### Comparing juvenile migration and survival

The premature break-ups in the winter of 2021/22 inadvertently introduced a quasi-experimental component to our study, where we had one group of juveniles known to have been forcibly and prematurely separated from their parents (the orphan group, *n* = 10), and another group (the reference group, *n* = 14) including all other juveniles caught that same winter. The reference group consisted of some juveniles which we knew to have remained with their parents based on GPS data (*n* = 4), but mostly juveniles for which we had no information on family status due to their parents not having been tagged (and no reason to assume loss of parents). We assumed that all juveniles in the reference group did not separate prematurely and remained with their parents after being caught.

We compared the timing of break-up, migration departure, migratory progress and survival until the next winter between juveniles from the orphan and the reference group. Migration departure was determined as the date of crossing 12.5°E, which roughly marks the eastern boundary of the Bewick’s swan core winter area (Linssen et al. 2024; Koffijberg et al. 2025). We measured migratory progress as the great-circle distance from the Ouse Washes in the United Kingdom (the location furthest from the breeding grounds reached by any of the birds). We used this measure because the migratory route of Bewick’s swans tracks largely eastward for the first half before taking a more northerly direction, making latitude a less informative measure. To determine survival until the next winter, we checked for tracking data from the juveniles in the following winters, or alternatively whether any resightings of the juveniles were logged during later winters in the international resighting database www.geese.org (Ebbinge et al. 2020; now defunct and integrated into https://submit.cr-birding.org/). Due to the high resighting probability (91% annually) of marked Bewick’s swans (Wood et al. 2018; Linssen et al. 2024), we are confident that the combination of tracking and resighting data gave us a reliable assessment of survival.

It was not possible to record data blindly for our study, because it involved focal animals in the field.

## Results

### Family break-up

**Fig. 2:**
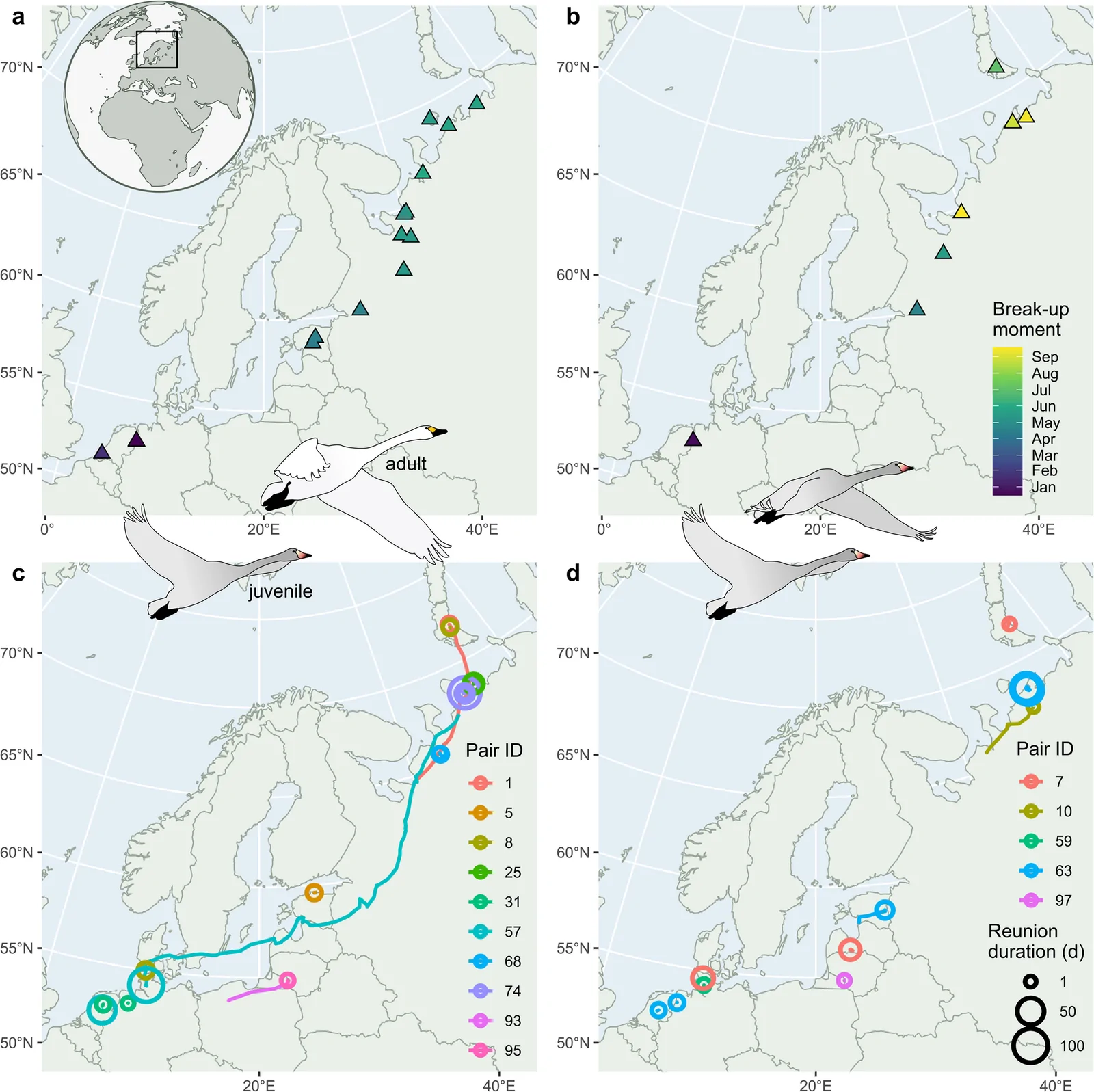
**a**,** b)** Bewick’s swan family break-up between **(a)** parent and offspring (*n =* 25 break-ups, of which 11 in the wintering range) and **(b)** siblings (*n =* 13 break-ups, of which 6 in the wintering range) during the first winter season, first northward migration or second summer season of the young birds. Colour denotes the month of breaking up. **c**,** d)** Reunions between **(c)** parent and offspring (*n =* 13 reunions in total among 16 pairs) and **(d)** siblings (*n =* 13 reunions in total among 13 pairs). Colours denote pair IDs (see Supplementary Information 1 Table S2, S3) and the symbol size denotes the reunion duration. Lines show the journeys that pairs travelled while reunited (either northward or southward); absence of a line means that the birds stayed in one location while reunited, and that the association ended with either bird leaving the site. Fig. created using RStudio (R Core Team 2023) and Inkscape

We GPS-tracked 18 parent-offspring pairs, of which we derived 16 break-ups (26 individuals from 10 families; Fig. 2a, Supplementary Information 1 Table S2; transmitter failure prevented us from establishing break-ups in the two remaining pairs). We determined nine additional break-ups from tagged juveniles that were parentless after capture (Supplementary Information 1 Table S1), yielding a total of 25 break-ups. In 14 cases, offspring separated from their parents along the second half of their first northward migration between Estonia and the breeding ground, from 10 April to 25 May. They were at that point approximately 9–11 months old (Supplementary Information 1 Table S2). In the other 11 cases, parent-offspring pairs broke up during their first midwinter. Seven of these early break-ups were due to the extreme roost disturbance by hunters: for example, in one family unit the father (047T) and daughter (036T) got separated (Fig. 1b) and the mother (027T) was killed. The other four early break-ups were likely the result of separation from parents during the catch, since the parents of those juveniles were not caught. The offspring were at that time roughly half a year old.

We derived break-ups for 13 sibling pairs from 7 families (18 individuals) from distances between their tracks (Fig. 2b, Supplementary Information 1 Table S3). Siblings showed a wider range in the time of separating than parent-offspring pairs. Eight sibling pairs separated between 20 April and 18 September (at approximately 10 to 15 months of age), i.e., anywhere from the second half of the first northward migration to the start of the second southward migration. Additionally, six sibling pairs from three families (9 individuals) separated during their first midwinter (6 months of age), all separations caused by the same hunting disturbance.

### Family reunion

Four parent-offspring pairs and two sibling pairs were tracked for less than three months after break-up. They did not reunite within those months, but we considered the period too short to include in our assessment of reunions in later life. Of the 12 parent-offspring pairs that we followed for at least three months after break-up, 8 reunited at least once (13 reunions in total; Fig. 2c, Supplementary Information 1 Table S2). Reunions ranged from within one day to 97 days, with 7 reunions lasting a week or longer (Supplementary Information 1 Table S2), and they occurred throughout the migratory range (Fig. 2c). Of the 12 sibling pairs that we followed for at least three months after break-up, 5 reunited at least once (13 reunions in total; Fig. 2d, Supplementary Information 1 Table S3). Similar to parent-offspring pairs, reunions between siblings ranged from less than one day to 73 days with six reunions lasting a week or more, and they occurred throughout the migratory range. Five pairs of birds travelled jointly after reuniting (Fig. 2c, d), covering part of the migratory journey together.

One female offspring (353E) showed a particularly strong case of social bonding. She completed her first northward migration with her parents all the way to the breeding site at 67°N, where she separated. After having spent her full second winter again together with her parents (who at that time had two new young; HL pers. obs.), she remained with them also for her second northward migration (Fig. 1a). During her third winter, the GPS transmitter of her mother stopped working, preventing us from tracking their distance further. However, 353E was regularly observed with her parents throughout winter and even midway through spring migration on Lake Ilmen, Russia (P. Glazov, pers. comm.), strongly suggesting that she performed even her third northward migration with her parents. We also highlight again the special case of roost disturbance by hunters, in which the mother of a family was killed. One daughter (036T) became separated from her father (047T) and siblings (365E and 367E), the three of whom stayed together after the disturbance (Fig. 1b, c). 036T did not encounter her father again until almost two years later on Novaya Zemlya, when they reunited at the start of the southward migration. They migrated together for a week, before separating along the White Sea.

### Juvenile migration and survival

Ten GPS-tagged juveniles were established as orphaned during winter 2021/22, having lost both of their parents (Supplementary Information 1 Table S1). Only one of them (036T) joined a new family group on the wintering grounds: she travelled to the UK where she joined another tagged family (family ID 27, siblings 356E and 358E) from early February onwards. She followed them for two weeks in the UK and subsequently to northern Germany, where the non-kin association eventually dissolved in mid-March and 036T departed on migration on 26 March. The remaining nine juveniles did not form new family bonds on the wintering grounds but instead loosely associated with wintering flocks of conspecifics and/or resident mute swans. They lingered behind when other Bewick’s swans departed from the Netherlands (mostly in February) and northern Germany (mostly during the first half of March).

**Fig. 3:**
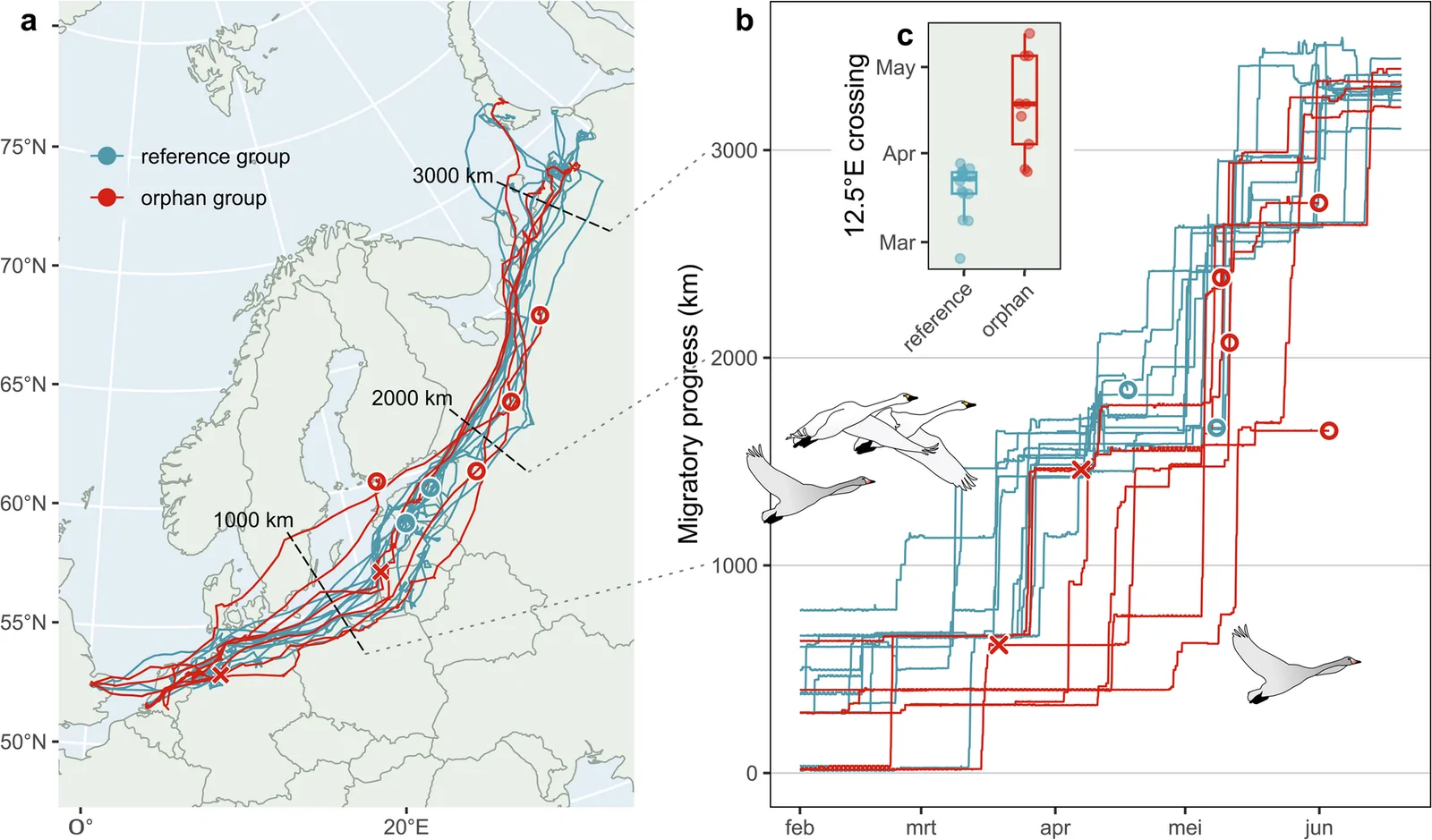
First northward migration of juveniles in 2022 that became orphaned during midwinter (red, *n* = 10) compared to juveniles with no indication of altered family status (reference group; blue, *n* = 14). **a)** migration tracks; **b)** migratory progress, measured as the distance (km) from the Ouse Washes in the United Kingdom (the location farthest from the breeding grounds reached by any of the birds); dashed transect lines in **a** indicate 1,000 km progress intervals for comparison. Circles indicate final track locations for individuals that subsequently died in the northern part of the range (four orphans and two from reference group); crosses indicate recorded deaths (two orphans). **c**) date of crossing 12.5°E, used as a proxy for migration departure. Boxes indicate the inter quartile range (IQR), with the central line depicting the median and the whiskers extending to 1.5*IQR. Fig. created using RStudio (R Core Team 2023) and Inkscape

Juveniles from the reference group, assumed to be under parental guidance, crossed 12.5°E between 23 February and 28 March (Fig. 3c). All but one of the nine orphans, which died prematurely on a night roost, eventually departed on migration without the guidance of any adults, crossing 12.5°E on average 29 days later (between 25 March and 12 May; two-sample t-test *t* = -4.63, df = 10.90, *p* < 0.001; Fig. 3c), when virtually the entire population had already left the winter range (pers. obs.; Nuijten et al. 2014, 2020b). Orphans generally headed northeast along a similar path, although along a broader front (Fig. 3a). Only four survived until the next winter; one died in Latvia in April and the remaining four sent their last GPS fix flying off the GSM network heading north, but the transmitters were never detected again, nor were the birds ever resighted in later winters, indicating that they died in the northern half of their range (Fig. 3a, b).

Survival until the next winter of the orphaned juveniles (4 out of 10, or 40%) was substantially lower than that of GPS-tagged juveniles in the reference group during that same winter of 2021/22 (12 out of 14, or 86%; Fisher’s exact test odds ratio = 0.124, 95% CI: 0.009–1.046, *p* = 0.032).

## Discussion

Using uniquely integrated tracking and genetic datasets from migratory Bewick’s swan families, our study reveals detailed patterns of family cohesion, break-up and subsequent reunions. Parents guided their offspring well into the second half of their first northward migration, when the offspring were approaching one year of age. In all cases where families broke up earlier, dissolution occurred on the wintering grounds due to disturbance events and crucially, led to delayed migration and lower survival of the juveniles. Our study empirically shows that extended parental care is not merely beneficial, but vital to young family-living birds.

Various studies found that parental guidance in waterfowl often ceases within the first winter (Jonker et al. 2011; Gupte et al. 2019). In a GPS-tracking study of greater white-fronted geese, many juveniles became independent during winter, presumably as a result of disturbance as judged from movement patterns preceding the moment of break-up (Gupte et al. 2019). In our study, cases of parent-offspring or sibling separations during the first winter, when offspring were roughly six months old, were caused by known or likely disturbance events. Moreover, all other break-ups occurred well outside the winter range, at least as far underway as Estonia, or halfway into the offspring’s first northward migration. At this point, offspring were approximately ten or eleven months old and had completed almost two migratory journeys in the company of their parents. This implies that Bewick’s swans normally or preferably sustain family bonds until well along their first northward migration, and that earlier break-ups are unintended and disadvantageous to either or both the offspring and their parents.

The frequent reunions observed between related birds further illustrate the importance of family bonds established during early life (Rees 2006a), with some offspring continuing to spend parts of subsequent winter seasons and migrations with their parents. Such reunions can extend beyond the second southward migration, as seen in swan 353E (Fig. 1a), and have been observed to an even greater extent in greater white-fronted geese, where familial associations lasting up to 13 years (Warren et al. 1993) and spanning three generations (Wilson et al. 2025) have been documented. Like many migratory species, swans rely on specific roosting and stopover sites that they use for extended periods (Nolet 2006; Nuijten et al. 2014; Nuijten and Nolet 2020). This increases the likelihood of relatives encountering each other during staging episodes, but that alone does not explain the tendency to remain together upon the encounter. Actively spending time together likely provides social benefits such as better access to food patches (Klaassen et al. 2006) or shared information on foraging or travel opportunities (Németh and Moore 2007; Bijleveld et al. 2010; Loonstra et al. 2023). But because such information exchange could in principle occur just as well among unrelated individuals, it seems that relatives reunite and travel together out of an intrinsic preference for related or familiar companions.

All but one of the juveniles that became inadvertently separated from their parents remained in the wintering areas longer than their conspecifics. Although they initially associated with wintering flocks of unrelated Bewick’s swans, they remained behind as those conspecifics departed on northward migration. Even more strikingly, they eventually departed independently after the entire population had already left, generally heading along a similar route. Except for 036T, who joined another GPS-tagged family, we do not know whether the juveniles reassociated with other Bewick’s swans after having left the Netherlands and Germany, but it seems plausible that the other three that survived until the following winter, may have done so by joining migrating flocks during later stages of their spring journey. Although our results show that first-wintering birds can closely associate with non-kin after loss of parents, they suggest that, in general, juveniles strongly rely on their own parents, rather than unrelated conspecifics for learning migratory performance. Nonetheless, our findings indicate that first-winter birds possess some knowledge of their migratory route and approximate migratory timing, inherited and/or learned from the first southward migration guided by parents (Mueller et al. 2013; Kashetsky et al. 2021).

The prematurely separated juveniles showed lower survival than those in the reference group, which were assumed or known to have naturally remained with their parents. The 86% survival of the reference group aligns with earlier modelling studies on Bewick’s swans (Wood et al. 2018; Nuijten et al. 2020a), endorsing the robustness of our survival comparison. Because neither transmitters nor colour bands of the non-surviving separated juveniles were ever detected again, the time and location of their death and behaviour leading up to their presumed death remain unknown. However, it is likely that their high mortality in the summer season was a combination of direct and indirect effects of the premature separation from their parents. Juveniles generally migrate less efficiently or slower than adults across bird species, at least when they do not travel together (Mueller et al. 2013; Crysler et al. 2016; Rotics et al. 2016; Byholm et al. 2022). Juvenile geese have been shown to rely directly on their parents during migration by flying behind them, thereby reducing the energy costs of flight (Kölzsch et al. 2020). In addition, juveniles forage less efficiently than adults across bird species (Weathers and Sullivan 1989; Wunderle 1991; Inger et al. 2010; Morel et al. 2024) including Bewick’s swans (Nolet et al. 2014). Consequently, being under guidance and protection from parents when competing with conspecifics is known to benefit foraging efficiency and food intake in Bewick’s swans (Scott 1980; Earnst and Bart 1991) and other waterfowl (Scott 1984; Black and Owen 1989; Turcotte and Bédard 1989). In common kestrels (*Falco tinnunculus*), the duration of extended parental care was slightly positively correlated with offspring survival, although it was generally much shorter (averaging 15 days; López-Idiáquez et al. 2018). Similarly, juvenile Caspian terns (*Hydroprogne caspia*) that lost a parent prematurely during early migration all died due to predation (*n* = 4; Byholm et al. 2022), although the birds did not migrate in family groups but generally a single chick travelled with a single parent. Predation risk is substantially smaller for the large-bodied Bewick’s swan, making it a less likely cause of death than the indirect accumulated effects of parent loss on energy maintenance. In greater white-fronted geese, survival of birds associating with their parents in winter was higher across ages 2–7, although the difference was less drastic than in our study (roughly 10%; Weegman et al. 2016). Our results indicate that the benefits of extended parental care are important to migratory, family-living birds to such an extent that they can critically affect offspring migration and survival well beyond the first half year of life.

Disturbance was the only confirmed cause of premature family break-up in our study, in the most severe cases through the death of family members. Bewick’s swans constitute one of the smallest and most rapidly declining waterfowl populations in the Western Palearctic (Rees and Beekman 2010; Beekman et al. 2019; Rees et al. 2019; Koffijberg et al. 2025). Although they are formally protected against hunting and disturbance across the flyway, hunting still occurs widely (Newth et al. 2011, 2022; Nagy et al. 2012; Buij et al. 2026). For many other waterfowl species, notably geese, disturbance is however deliberately applied at broad scales as a management tool (Madsen 1995; Percival et al. 1997; Tombre et al. 2013). We emphasise that disturbance, beyond its immediate effects (whether intended or not) on displacement and energy expenditure (e.g., Madsen 1995; Béchet et al. 2004; Nolet et al. 2016; Linssen et al. 2019; Hoekstra et al. 2024), can have long-lasting consequences for family-living birds by damaging existing family structures, including in species targeted by disturbance-based population management. For species under stricter protection, such as the Bewick’s swan, consistent efforts must be made to minimise disturbance during the highly social wintering period and ensure the availability of sufficient undisturbed refuges. Moreover, our study suggests that scientific catching efforts can also disrupt family cohesion, underscoring the need for careful consideration of animal tagging and aiming for least-disruptive capture strategies. Automated detection of disturbance events, or even of family break-up, directly from tracking data is an important avenue for future research (see Gupte et al. 2019), though validation against extensive field observations will be essential (de Knegt et al. 2021).

Social learning is ubiquitous in the animal kingdom, occurring not only between kin but also among unrelated individuals (Heyes 1994; van Schaik 2010; Aplin 2019; Aikens et al. 2022; Newton 2024). Among migratory birds, young whooping cranes (*Grus americana*) famously learn migration from older and more experienced, but not necessarily related, flock members (Mueller et al. 2013). Similarly, spoonbills migrate in large mixed-age flocks in which juveniles are no longer accompanied by their parents (Lok et al. 2025), whereas the first southward journey of black-tailed godwits (*Limosa limosa*) is shaped by interactions with inexperienced conspecifics in the absence of adults (Verhoeven et al. 2022; Loonstra et al. 2023). In light of this, it is remarkable that all but one of the orphaned swans in our study seemed unable or unwilling to compensate for the loss of their family bonds by closely associating with non-kin, at least in the wintering area where we observed them, despite the ample presence of conspecifics in their proximity. In contrast to sandhill cranes (*Grus canadensis*), where the duration of family associations was found not to impact survival (Hayes and Barzen 2016), a much higher proportion of the separated swans died within a year. While effects of prolonged stress from the disturbance events that disrupted their families cannot be ruled out, this pattern may reflect interspecies differences in the specific social environment evolved to facilitate social learning. While species like cranes, spoonbills and godwits tend to generally rely on broad social networks to learn migration, our study suggests that species with strong and extended family bonds, such as the Bewick’s swan, may rely on a much more closed network composed primarily of kin, with family life serving as the primary pathway for social learning. As we are only beginning to unravel the complexity and diversity of social learning in animals, further elucidating the social environments in which learning occurs across species will be key to understanding how animal behaviour and migration are shaped.

## Supplementary Information

Below is the link to the electronic supplementary material.

Supplementary Material 1

Supplementary Material 2

## Data Availability

Data and code are available at Zenodo (Linssen et al. 2026). The raw DNA sequencing data are accessioned on the National Center for Biotechnology Information (NCBI) Sequence Read Archive (BioProject: PRJNA1290665, Biosample accessions: SAMN49931523-SAMN49931756).
