# Supplementary material for: Family life is critical for migration and survival in a long-lived social bird
Source: Behav Ecol Sociobiol. 2026 Aug 20;80(9):114. doi: 10.1007/s00265-026-03787-5 (PMC13493472; doi:10.1007/s00265-026-03787-5)
Supplement: Supplementary file 1 [file 265_2026_3787_MOESM1_ESM.pdf]

Supplementary Information 1 to "Family life is critical for migration and survival in a long-lived social bird": supplementary figures and tables

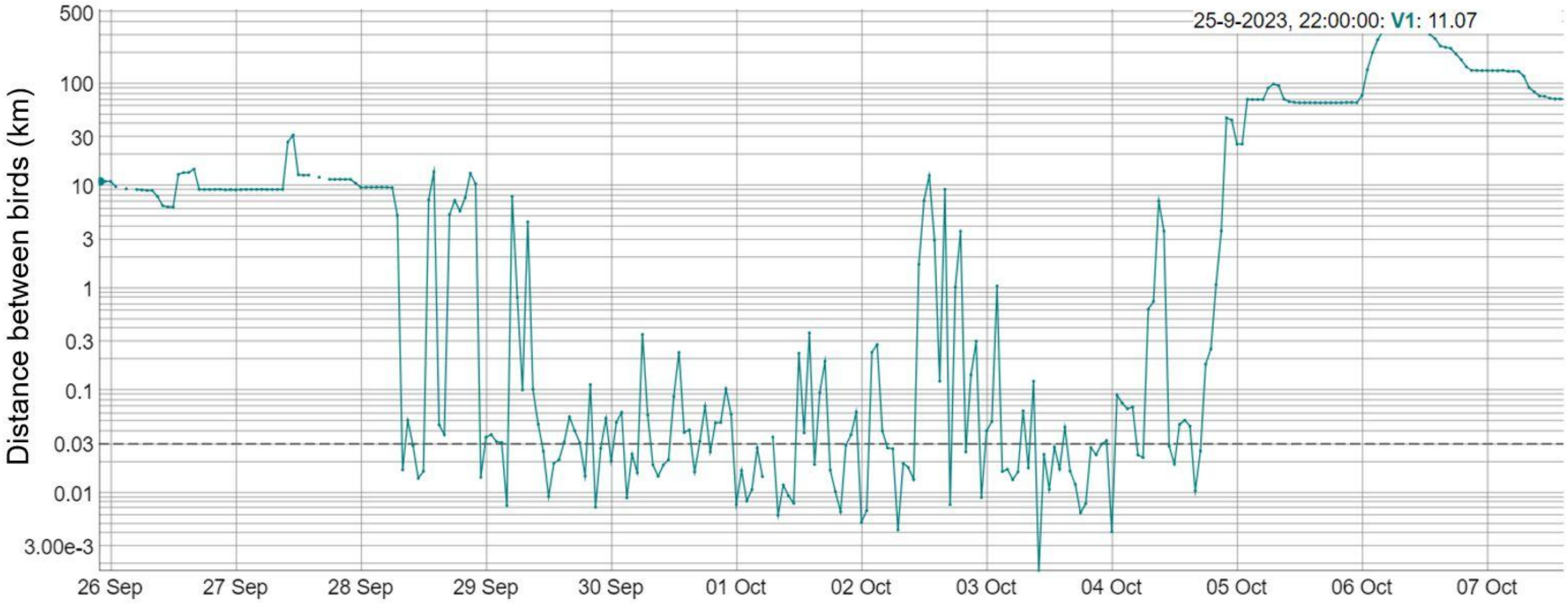

Fig S1 Example of a reunion between father (047T) and daughter (036T) in Sept-Oct 2023. They encounter each other on Novaya Zemlya and travel south together for one week (see Fig. 1b). The grey dashed line at 30 m indicates the threshold used for assessing break-ups and reunions.

Table S1 Overview of GPS-tagged juveniles in the orphan group (having prematurely lost their parents in the winter range) and reference group. Migration departure was determined as the date of crossing 12.5°E. 036T was the only orphan found to join GPS-tagged non-kin on the wintering grounds (siblings 356E and 358E). Absence of a family ID means that we did not catch any related individuals of the juvenile.

Juvenile	Family ID	Group	Established orphan status through…	Migration departure (12.5°E crossing)	Survival until next winter	Notes on mortality
365E	1	Reference	-	23 Mar	1	
367E	1	Reference	-	23 Mar	1	
035T	9	Reference	-	28 Mar	1	
361E	19	Reference	-	8 Mar	1	
362E	21	Reference	-	18 Mar	1	
364E	21	Reference	-	23 Feb	1	
356E	27	Reference	-	25 Mar	1	
358E	27	Reference	-	25 Mar	0	Never returned from breeding range
357E		Reference	-	17 Mar	1	
366E		Reference	-	8 Mar	1	
372E		Reference	-	22 Mar	1	
376E		Reference	-	26 Mar	0	Never returned from breeding range
380E		Reference	-	24 Mar	1	
381E		Reference	-	18 Mar	1	
036T	1	Orphan	Combining GPS tracking data with tracks of father (See Table S2) and retrieving dead body of mother (027T)	26 Mar	1	
049T	28	Orphan	Field observations on winter grounds	18 Apr	1	

363E	28	Orphan	Field observations on winter grounds	18 Apr	0	Never returned from breeding range
028T	28	Orphan	Field observations on winter grounds	-	0	Died on night roost in the winter range
032T	28	Orphan	Field observations on winter grounds	25 Mar	0	Died at staging site in Latvia
368E		Orphan	Field observations on winter grounds	12 May	0	Never returned from breeding range
377E		Orphan	Field observations on winter grounds	4 May	0	Never returned from breeding range
378E		Orphan	Field observations on winter grounds	4 May	0	Never returned from breeding range
382E		Orphan	Field observations on winter grounds	4 Apr	1	
383E		Orphan	Field observations on winter grounds	13 Apr	1	

Table S2 Overview of recorded break-ups and reunions between pairs of Bewick's swan parent and offspring. F = female, M = male. Negative day of year (DOY) values indicate days before 1 January. *approximate age of offspring at the occurrence of break-up, assuming a hatching date of 1 July. The two break-ups at six months were caused by roost disturbance (along with eight more premature break-ups not included in this table, which were derived from single, unpaired juvenile tracks combined with field observations, see main text). In the case of father 044T and daughter 364E (pair ID 21), we have no information on the identity of the mother, meaning that we cannot establish whether 364E became parentless or remained with her mother after the break-up. 364E is therefore not included in the survival calculation of parentless juveniles.

Family ID	Pair ID	Parent (sex)	Offspring	Break-up date	Break-up DOY	Break-up latitude (°)	Break-up longitude (°)	Offspring age at break-up (months)*	Time tracked after break-up (d)	# times within 30 m	# reunions	Reunion durations (d)
1	1	047T (M)	036T (F)	20 Dec '21	-11	52.73	7.16	6	1176	3	1	7
1	5	047T (M)	365E (F)	11 May '22	131	63.37	40.02	10	1033	1	1	6
1	8	047T (M)	367E (M)	11 May '22	131	63.37	40.02	10	1034	2	2	6, >8
5	28	230E (F)	228E (M)	20 Apr '17	110	60.11	31.76	10	3	0	0	
5	31	230E (F)	237E (M)	17 May '17	137	63.63	38.88	11	1562	3	2	2, 4
7	53	223E (F)	224E (F)	17 May '18	137	69.11	48.67	11	174	0	0	
8	55	350E (F)	352E (F)	07 May '21	127	64.69	40.07	10	359	0	0	
8	57	350E (F)	353E (F)	07 May '21	127	64.69	40.07	10	571	2	2	50, 97
8	60	350E (F)	354E (F)	07 May '21	127	64.69	40.07	10	571	0	0	
9	65	013T (M)	035T (M)	14 May '22	134	64.77	40.35	10	712	1	0	
12	74	208E (F)	207E (M)	25 May '17	145	68.33	51.00	11	706	4	3	77, 13, 3
15	81	248E (F)	249E (M)	10 Apr '19	100	58.63	25.69	9	25	0	0	
16	85	255E (F)	257E (M)	15 Apr '20	106	58.93	26.06	9	41	0	0	
21	95	044T (M)	364E (F)	23 Dec '21	-8	52.73	7.15	6	475	1	1	3
21	93	044T (M)	362E (F)	13 May '22	133	61.72	38.03	10	334	1	1	3
22	98	056T (M)	053T (F)	23 May '23	143	68.59	56.76	11	55	0	0	

Table S3 Overview of recorded break-ups and reunions between pairs of Bewick's swan siblings. F = female, M = male. Negative day of year (DOY) values indicate days before 1 January. *approximate age of siblings at the occurrence break-up, assuming a hatching date of 1 July. The seven break-ups at six months were caused by roost disturbance. Siblings 361E and 043T (family ID 19) separated prematurely on 18 December, meaning that one or both may have become orphaned. However, as their parents were not tagged and no field observations support this, they are retained in the reference group.

Family ID	Pair ID	Sibling 1	Sibling 2	Break-up date	Break-up DOY	Break-up latitude (°)	Break-up longitude (°)	Sibling age at break-up (months)*	Time tracked after break-up (d)	# times within 30 m	# reunions	Reunion durations (d)
1	4	036T (F)	365E (F)	21 Dec '21	-10	52.73	7.16	6	1176	0	0	
1	7	036T (F)	367E (M)	20 Dec '21	-11	52.73	7.16	6	1033	4	3	19, 2, 24
1	10	365E (F)	367E (M)	03 Jul '22	184	71.41	54.34	12	1034	4	2	<1, <1
3	20	359E (F)	360E (M)	13 Sep '23	256	68.17	54.66	14	107	0	0	
5	30	228E (M)	237E (M)	20 Apr '17	110	60.11	31.76	10	3	0	0	
8	59	352E (F)	353E (F)	18 Sep '21	261	64.76	40.14	15	1562	1	1	3
8	62	352E (F)	354E (F)	25 Aug '21	237	68.26	52.30	14	174	0	0	
8	63	353E (F)	354E (F)	28 Aug '21	240	68.30	52.31	14	359	10	6	10, 42, 4, 73, 5, 65
21	97	362E (F)	364E (F)	23 Dec '21	-8	52.73	7.15	6	571	4	1	5
28	111	032T (M)	028T (F)	23 Dec '21	-8	52.73	7.15	6	712	0	0	
28	114	032T (M)	363E (M)	23 Dec '21	-8	52.73	7.15	6	1038	0	0	
28	112	032T (M)	049T (F)	23 Dec '21	-8	52.73	7.15	6	706	0	0	
29	117	232E (M)	218E (M)	17 May '17	137	62.85	36.37	11	25	1	0	
