# Supplementary material for: Family life is critical for migration and survival in a long-lived social bird
Source: Behav Ecol Sociobiol. 2026 Aug 20;80(9):114. doi: 10.1007/s00265-026-03787-5 (PMC13493472; doi:10.1007/s00265-026-03787-5)

Supplementary Information 2 to “Family life is critical for migration and survival in a long-lived social bird”: Plots of hourly distances for all related bird pairs

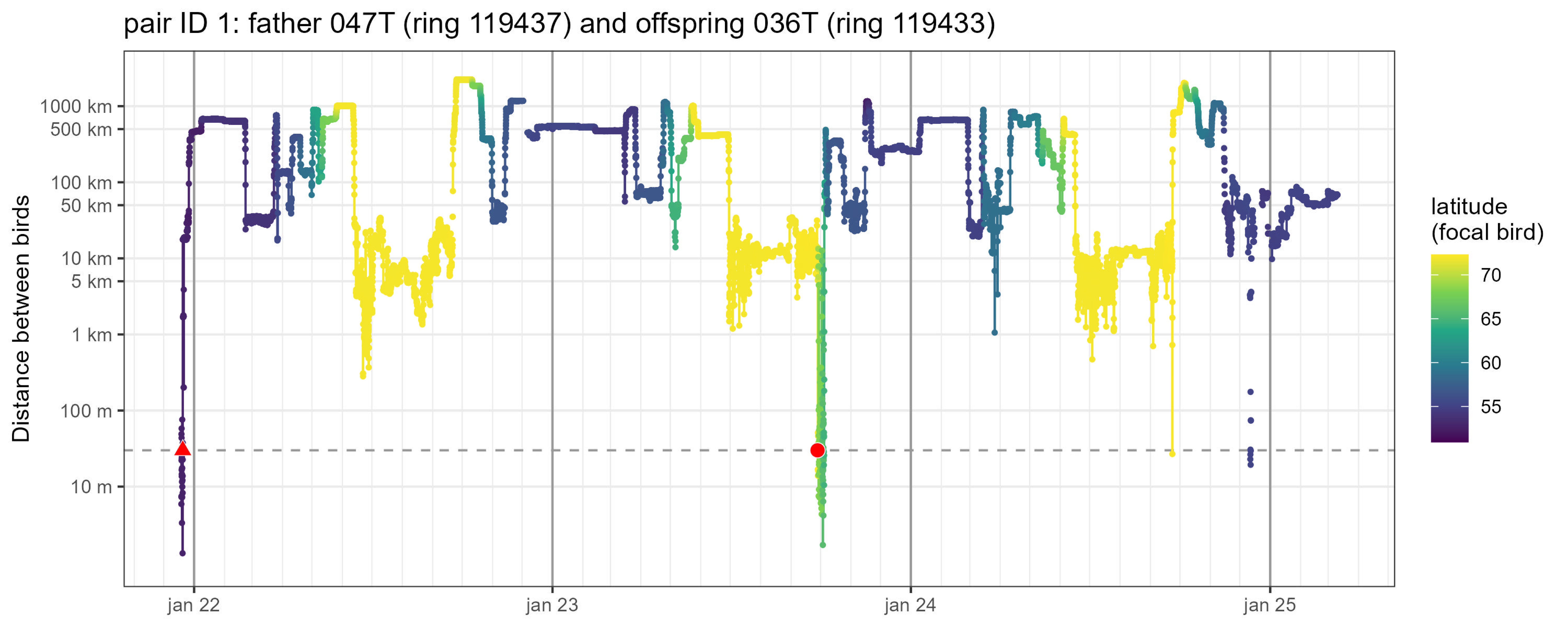

pair ID 1: father 047T (ring 119437) and offspring 036T (ring 119433)
Distance between birds
1000 km
500 km
100 km
50 km
10 km
5 km
1 km
100 m
10 m
jan 22
jan 23
jan 24
jan 25
latitude
(focal bird)
70
65
60
55

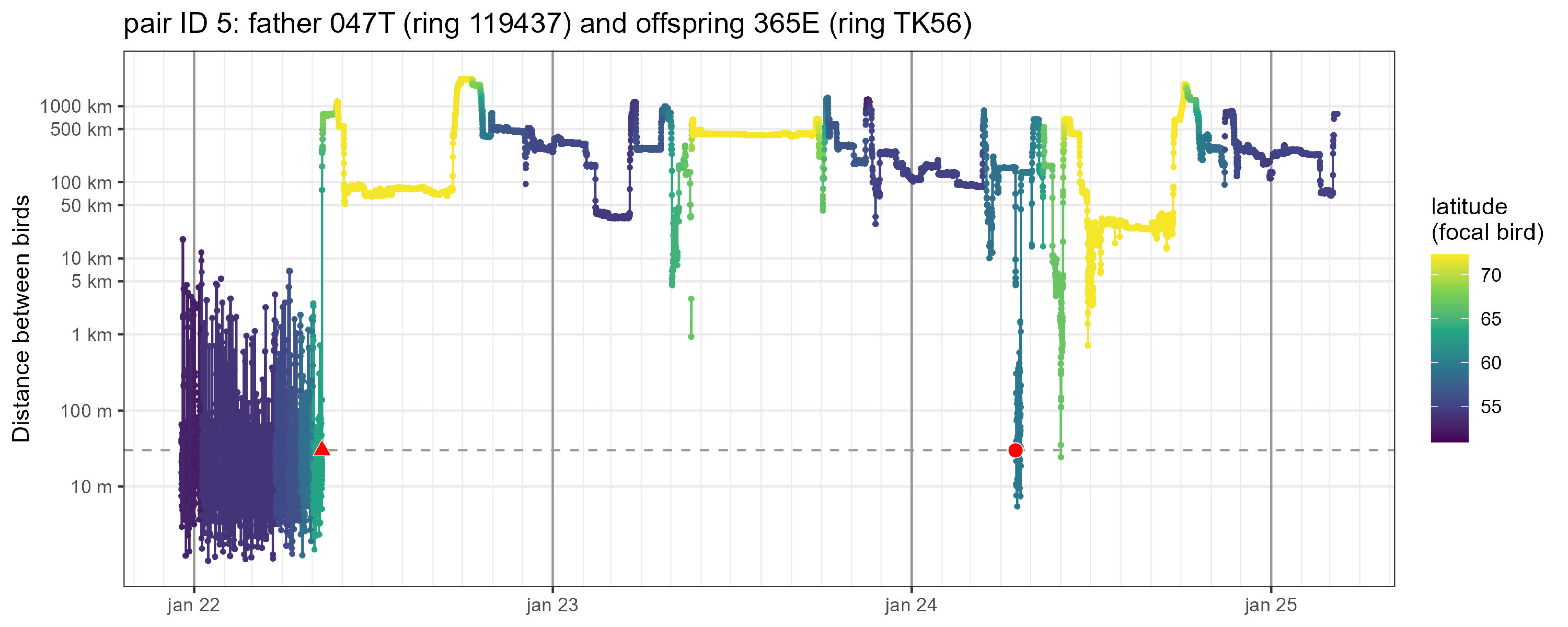
pair ID 5: father 047T (ring 119437) and offspring 365E (ring TK56)
Distance between birds
1000 km
500 km
100 km
50 km
10 km
5 km
1 km
100 m
10 m
jan 22
jan 23
jan 24
jan 25
latitude
(focal bird)
70
65
60
55

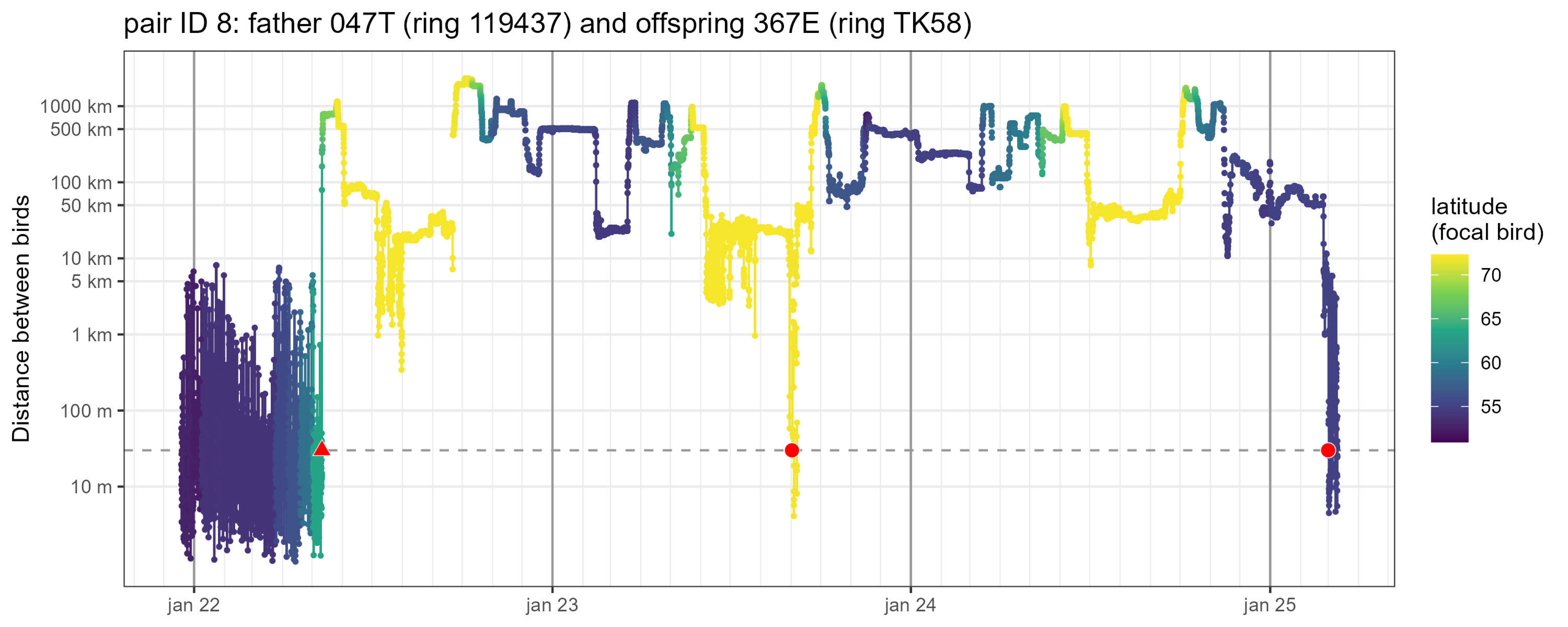
pair ID 8: father 047T (ring 119437) and offspring 367E (ring TK58)
Distance between birds
1000 km
500 km
100 km
50 km
10 km
5 km
1 km
100 m
10 m
jan 22
jan 23
jan 24
jan 25
latitude
(focal bird)
70
65
60
55

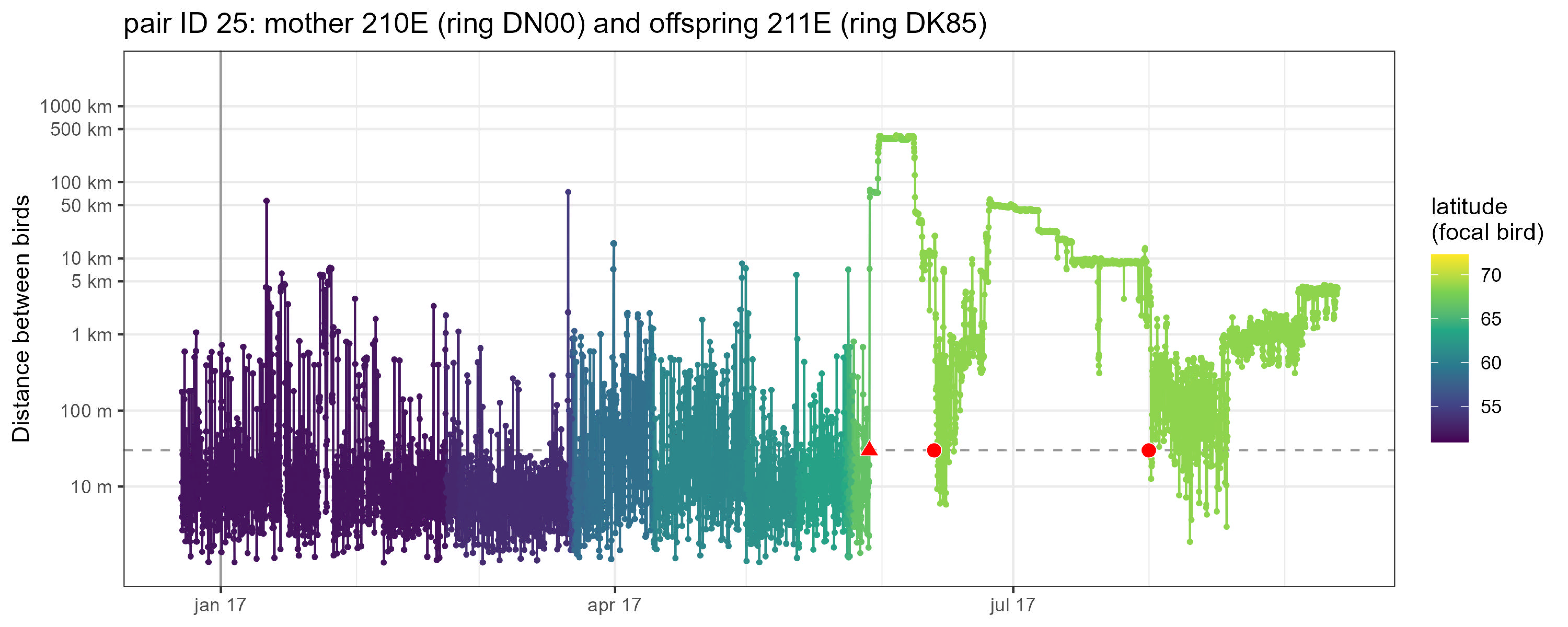

pair ID 25: mother 210E (ring DN00) and offspring 211E (ring DK85)
Distance between birds
1000 km
500 km
100 km
50 km
10 km
5 km
1 km
100 m
10 m
jan 17
apr 17
jul 17
latitude
(focal bird)
70
65
60
55

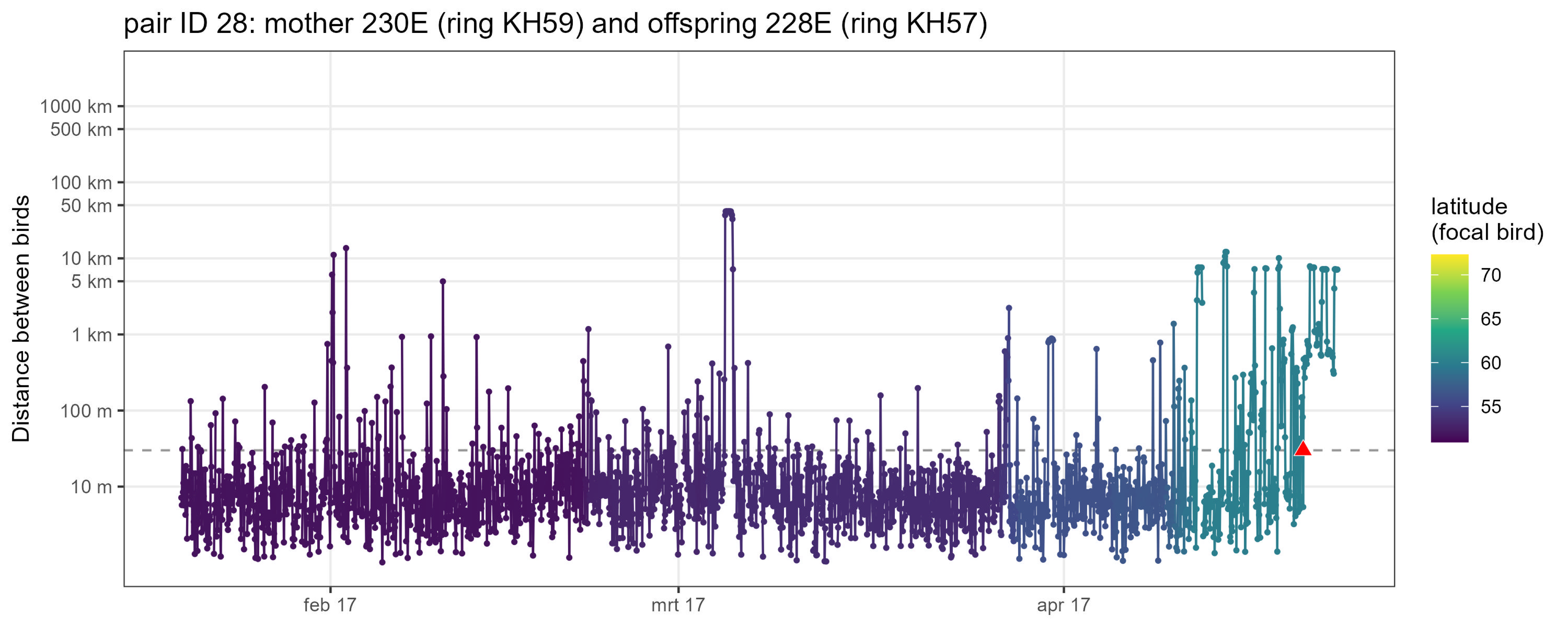

pair ID 28: mother 230E (ring KH59) and offspring 228E (ring KH57)
Distance between birds
1000 km
500 km
100 km
50 km
10 km
5 km
1 km
100 m
10 m
feb 17
mrt 17
apr 17
latitude
(focal bird)
70
65
60
55

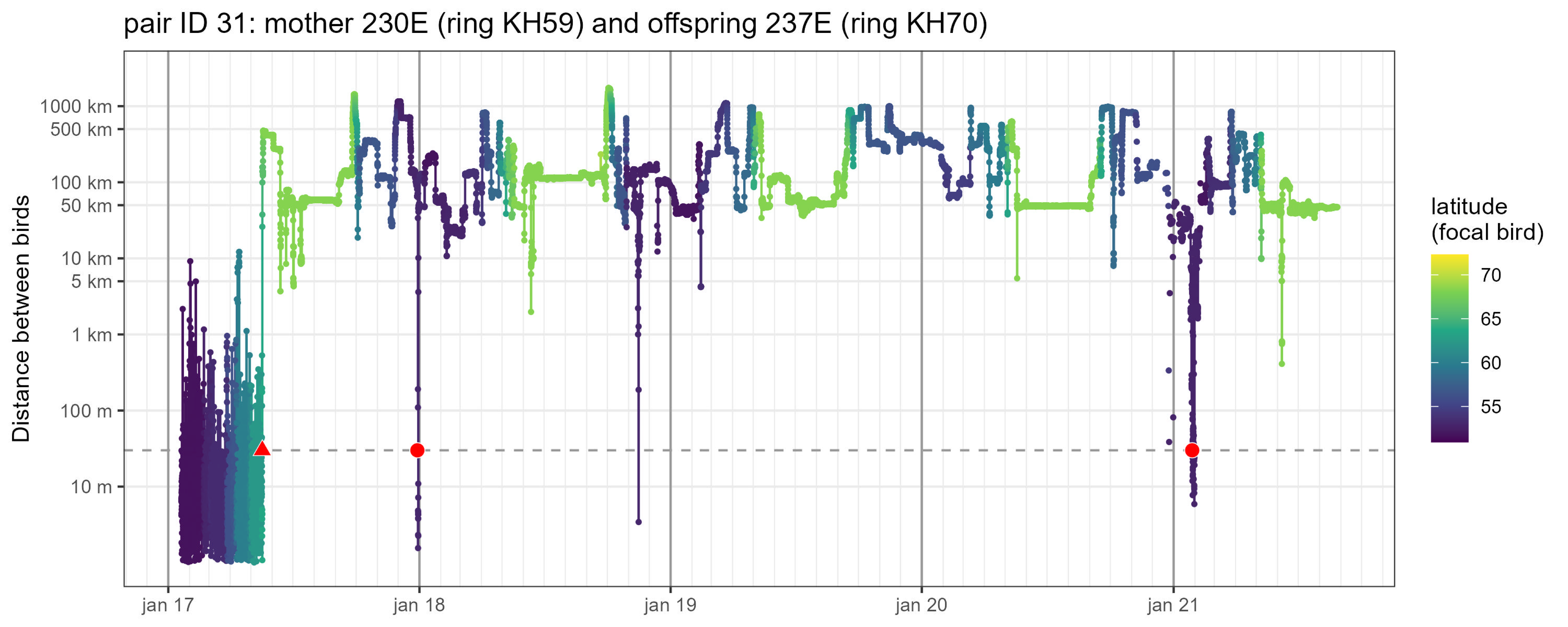
pair ID 31: mother 230E (ring KH59) and offspring 237E (ring KH70)
Distance between birds
1000 km
500 km
100 km
50 km
10 km
5 km
1 km
100 m
10 m
jan 17
jan 18
jan 19
jan 20
jan 21
latitude
(focal bird)
70
65
60
55

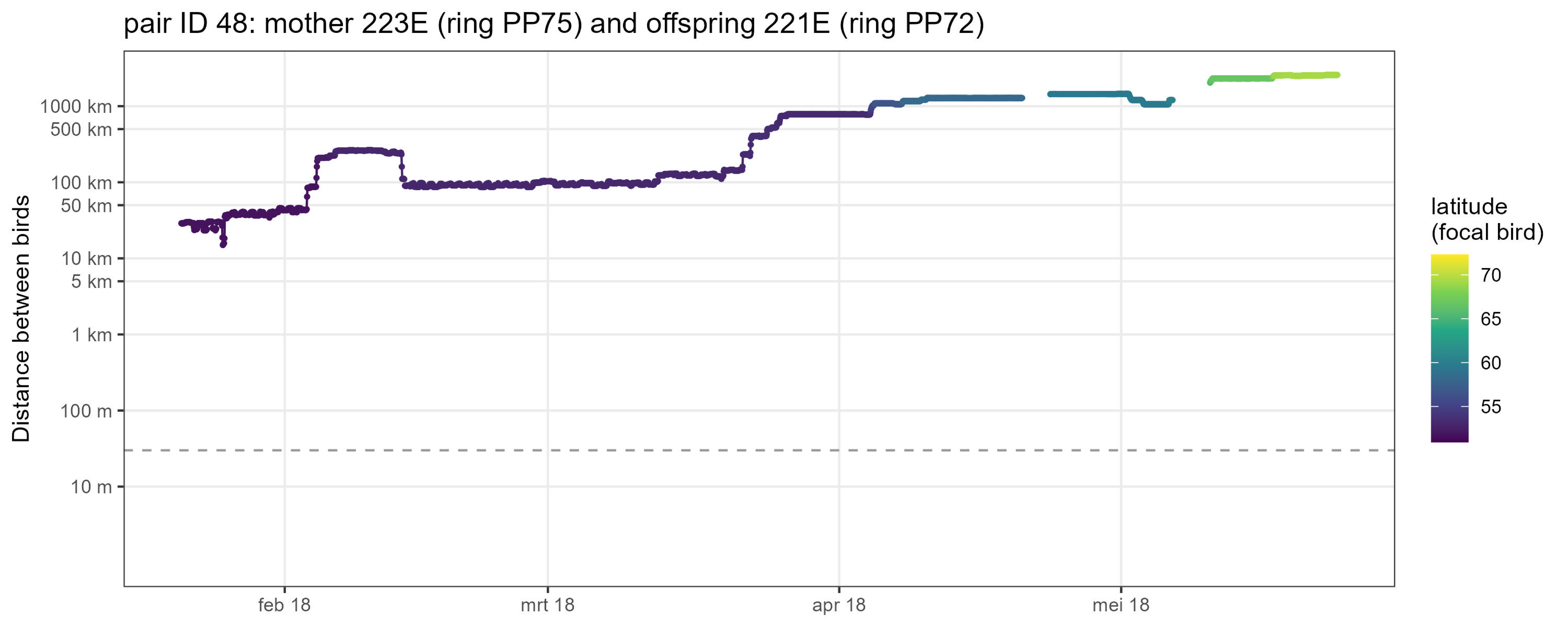
pair ID 48: mother 223E (ring PP75) and offspring 221E (ring PP72)
Distance between birds
1000 km
500 km
100 km
50 km
10 km
5 km
1 km
100 m
10 m
feb 18
mrt 18
apr 18
mei 18
latitude
(focal bird)
70
65
60
55

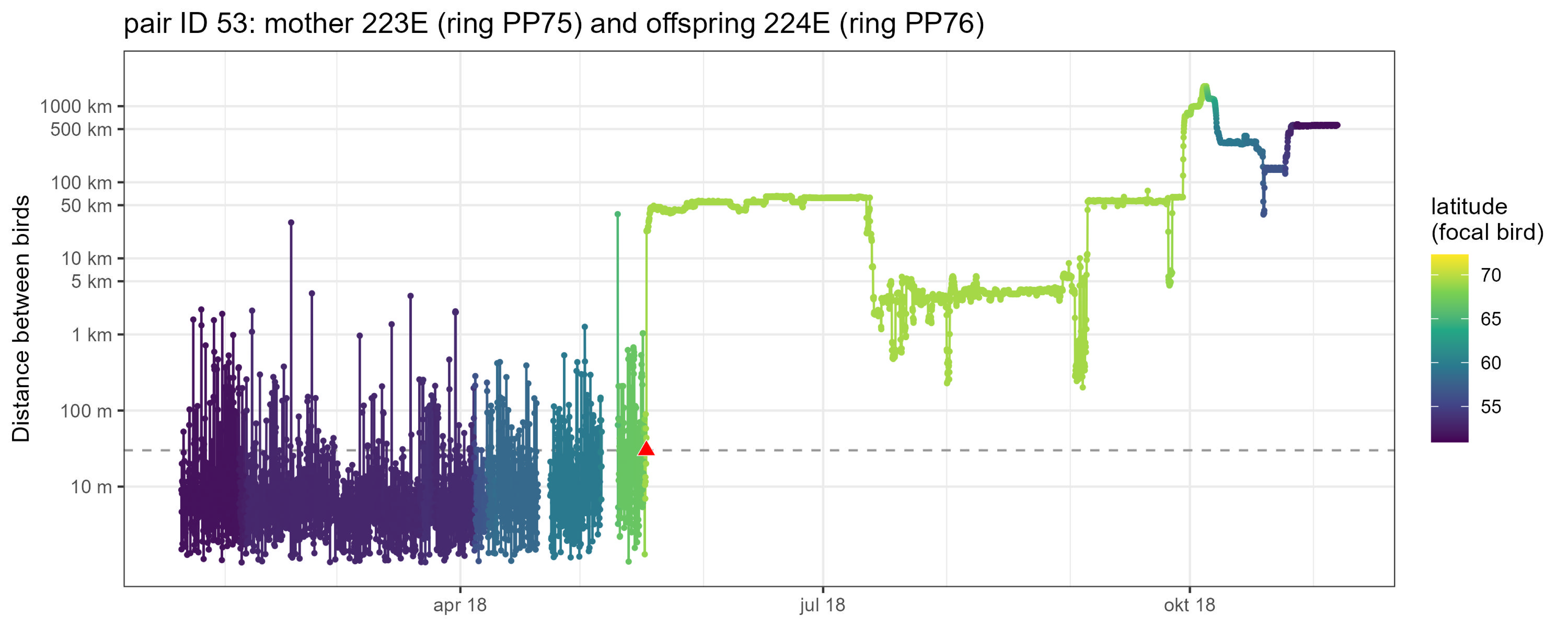

pair ID 53: mother 223E (ring PP75) and offspring 224E (ring PP76)
Distance between birds
1000 km
500 km
100 km
50 km
10 km
5 km
1 km
100 m
10 m
apr 18
jul 18
okt 18
latitude
(focal bird)
70
65
60
55

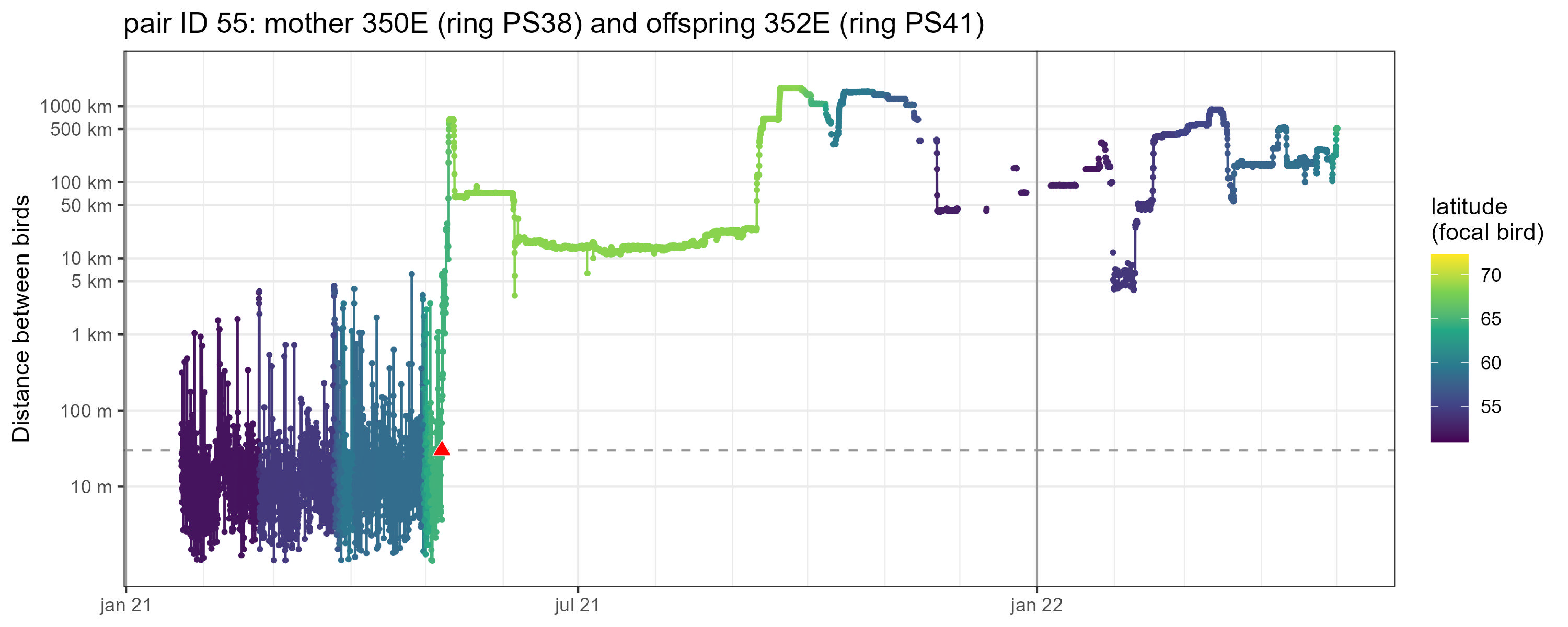

pair ID 55: mother 350E (ring PS38) and offspring 352E (ring PS41)
Distance between birds
1000 km
500 km
100 km
50 km
10 km
5 km
1 km
100 m
10 m
jan 21
jul 21
jan 22
latitude
(focal bird)
70
65
60
55

pair ID 57: mother 350E (ring PS38) and offspring 353E (ring PS42)

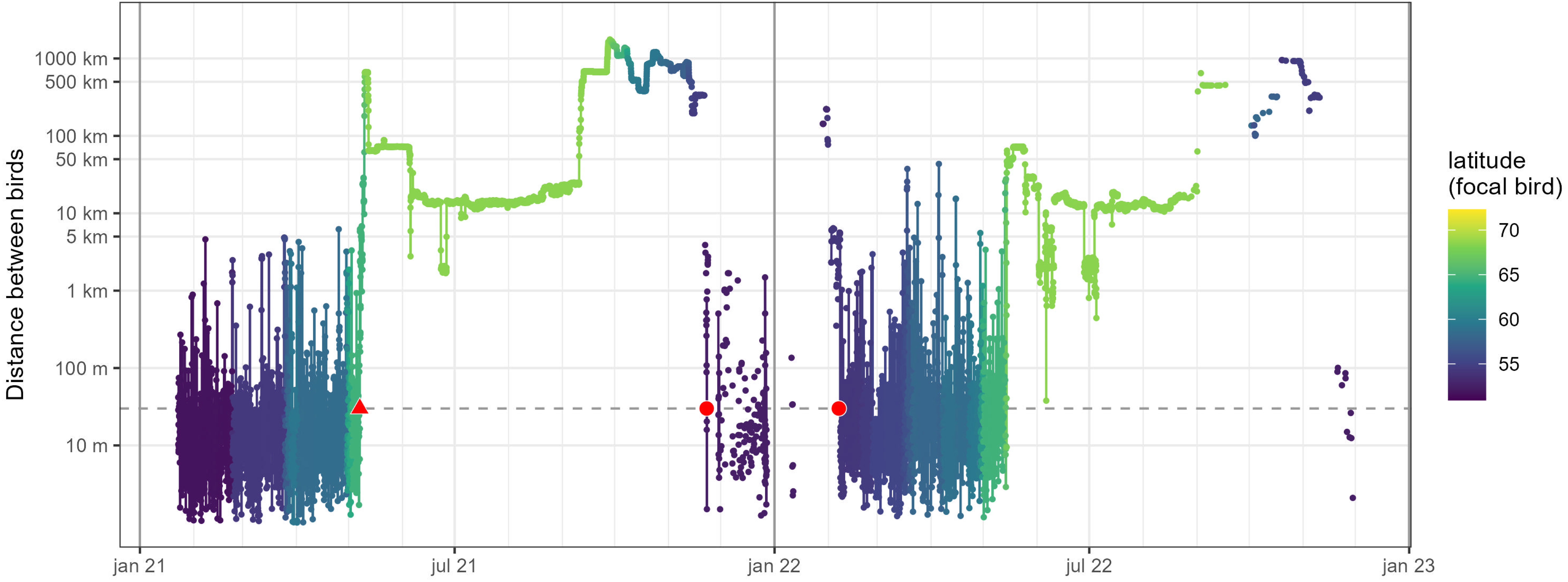

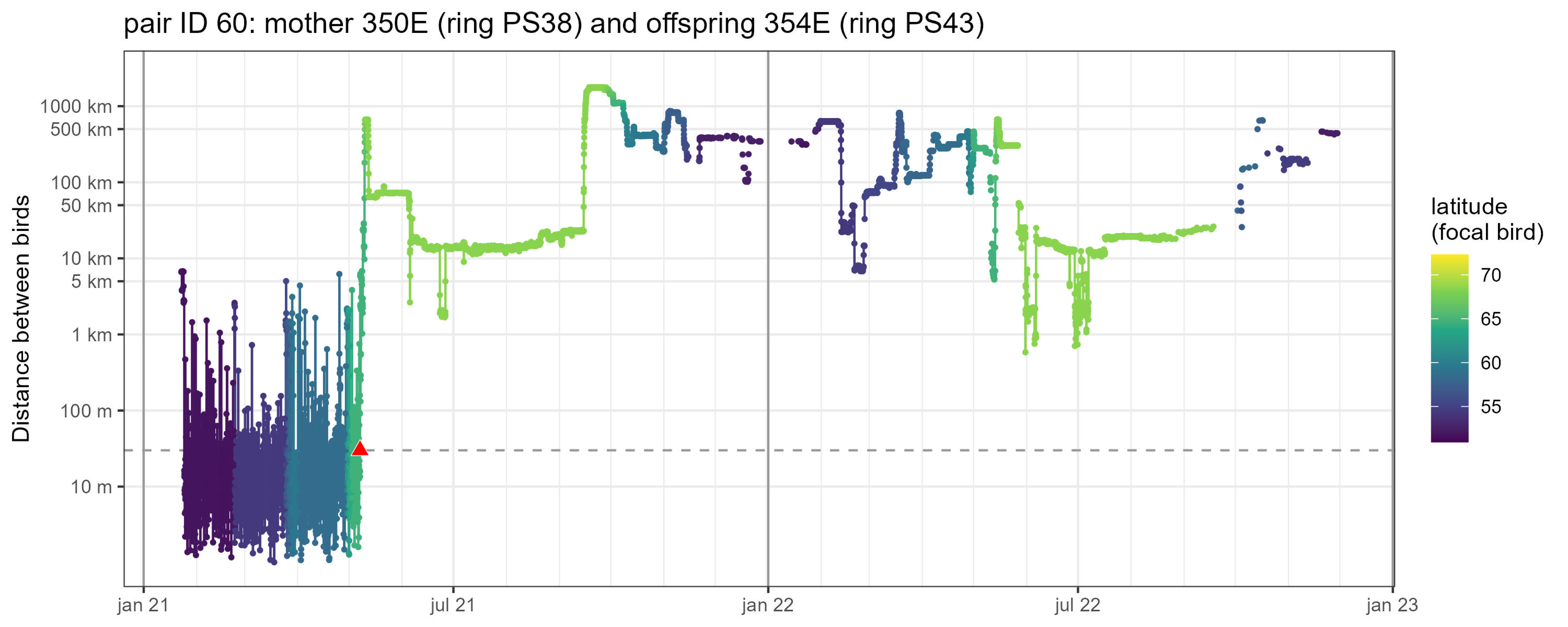
pair ID 60: mother 350E (ring PS38) and offspring 354E (ring PS43)
Distance between birds
1000 km
500 km
100 km
50 km
10 km
5 km
1 km
100 m
10 m
jan 21
jul 21
jan 22
jul 22
jan 23
latitude
(focal bird)
70
65
60
55

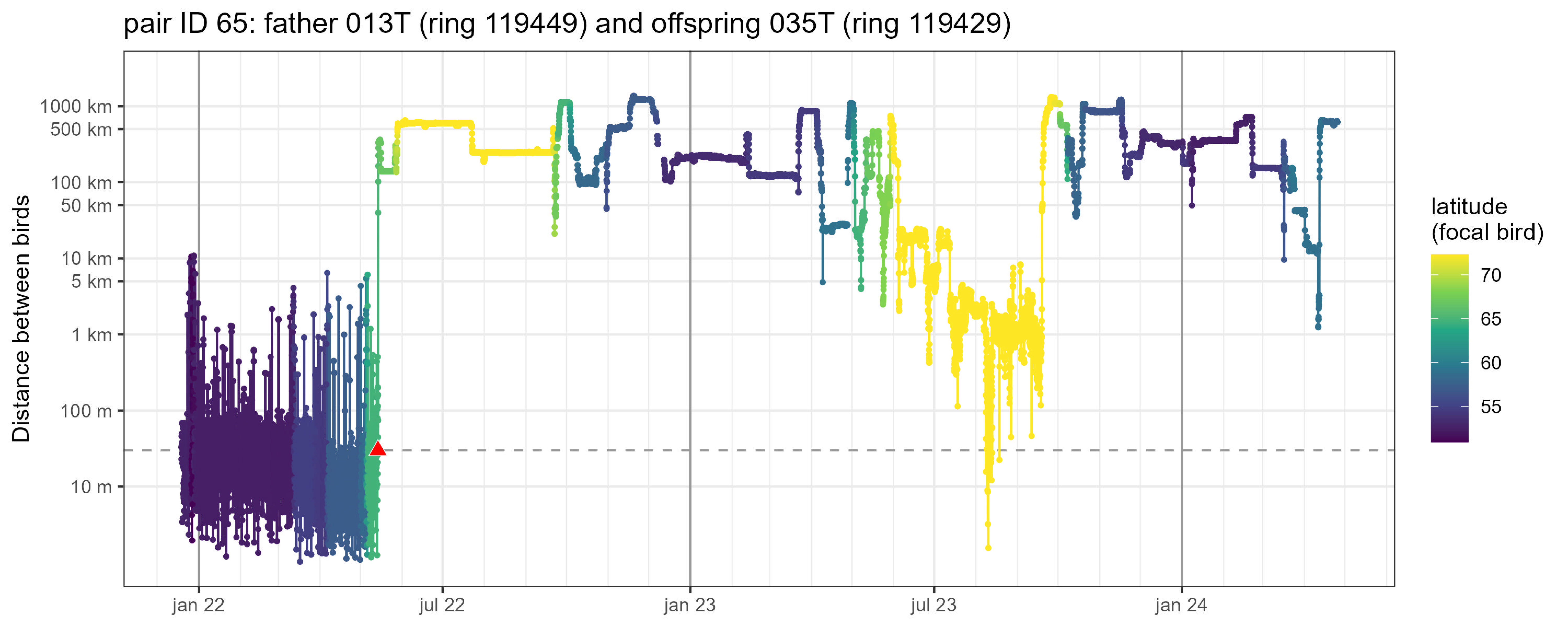

pair ID 65: father 013T (ring 119449) and offspring 035T (ring 119429)
Distance between birds
1000 km
500 km
100 km
50 km
10 km
5 km
1 km
100 m
10 m
jan 22
jul 22
jan 23
jul 23
jan 24
latitude (focal bird)
70
65
60
55

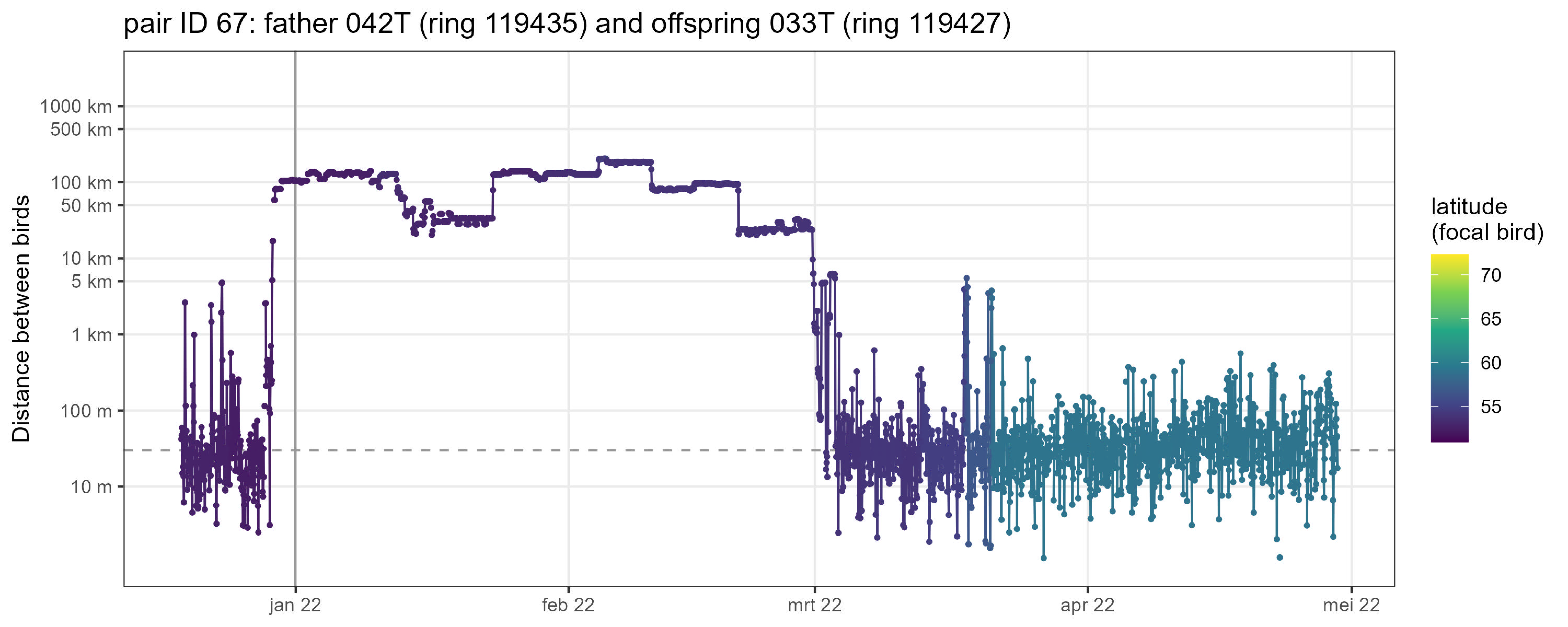
pair ID 67: father 042T (ring 119435) and offspring 033T (ring 119427)
Distance between birds
1000 km
500 km
100 km
50 km
10 km
5 km
1 km
100 m
10 m
jan 22
feb 22
mrt 22
apr 22
mei 22
latitude
(focal bird)
70
65
60
55

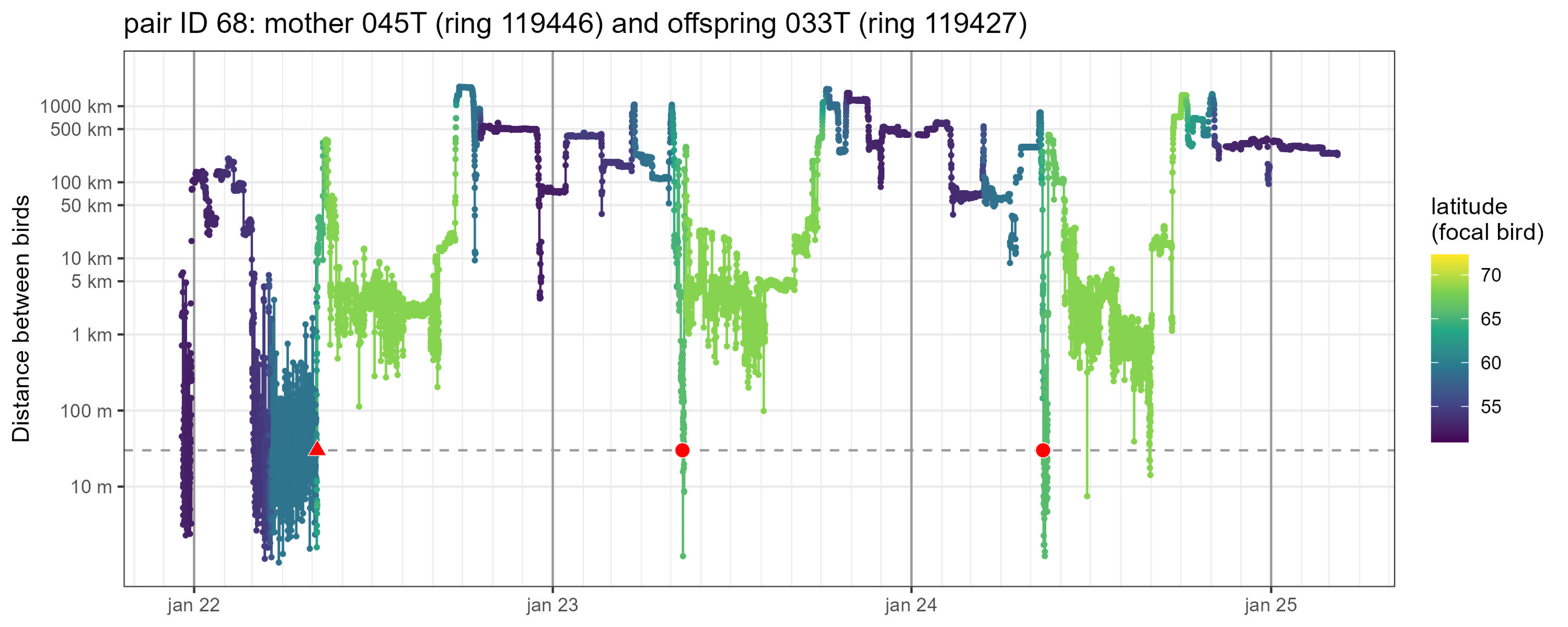

pair ID 68: mother 045T (ring 119446) and offspring 033T (ring 119427)
Distance between birds
1000 km
500 km
100 km
50 km
10 km
5 km
1 km
100 m
10 m
jan 22
jan 23
jan 24
jan 25
latitude
(focal bird)
70
65
60
55

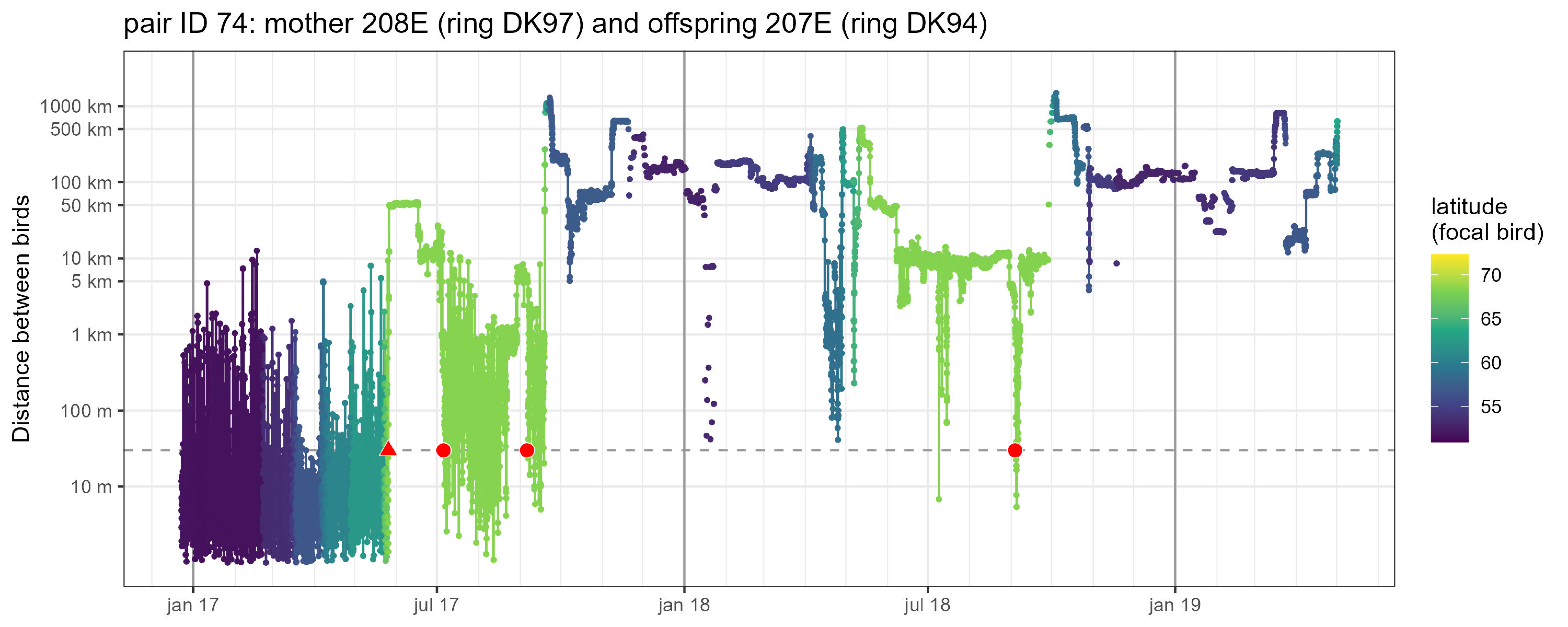
pair ID 74: mother 208E (ring DK97) and offspring 207E (ring DK94)
Distance between birds
1000 km
500 km
100 km
50 km
10 km
5 km
1 km
100 m
10 m
jan 17
jul 17
jan 18
jul 18
jan 19
latitude
(focal bird)
70
65
60
55

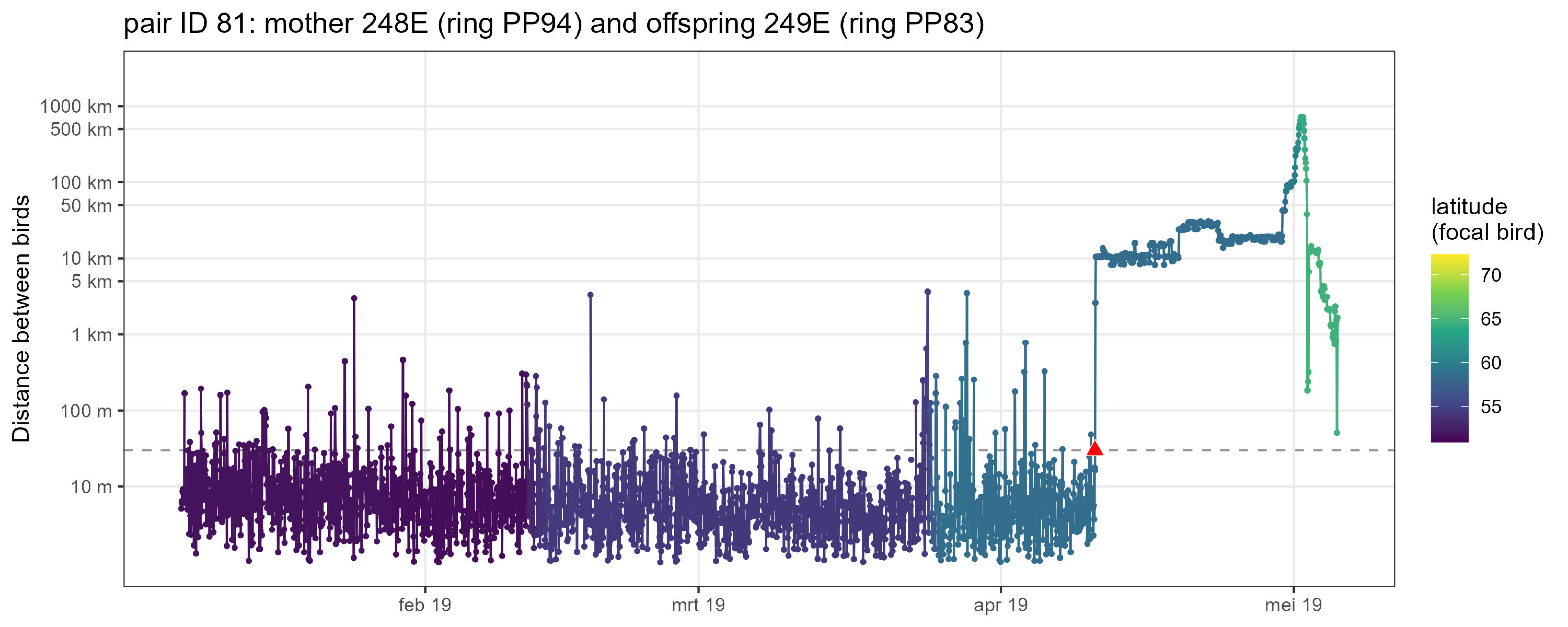

pair ID 81: mother 248E (ring PP94) and offspring 249E (ring PP83)
Distance between birds
1000 km
500 km
100 km
50 km
10 km
5 km
1 km
100 m
10 m
feb 19
mrt 19
apr 19
mei 19
latitude
(focal bird)
70
65
60
55

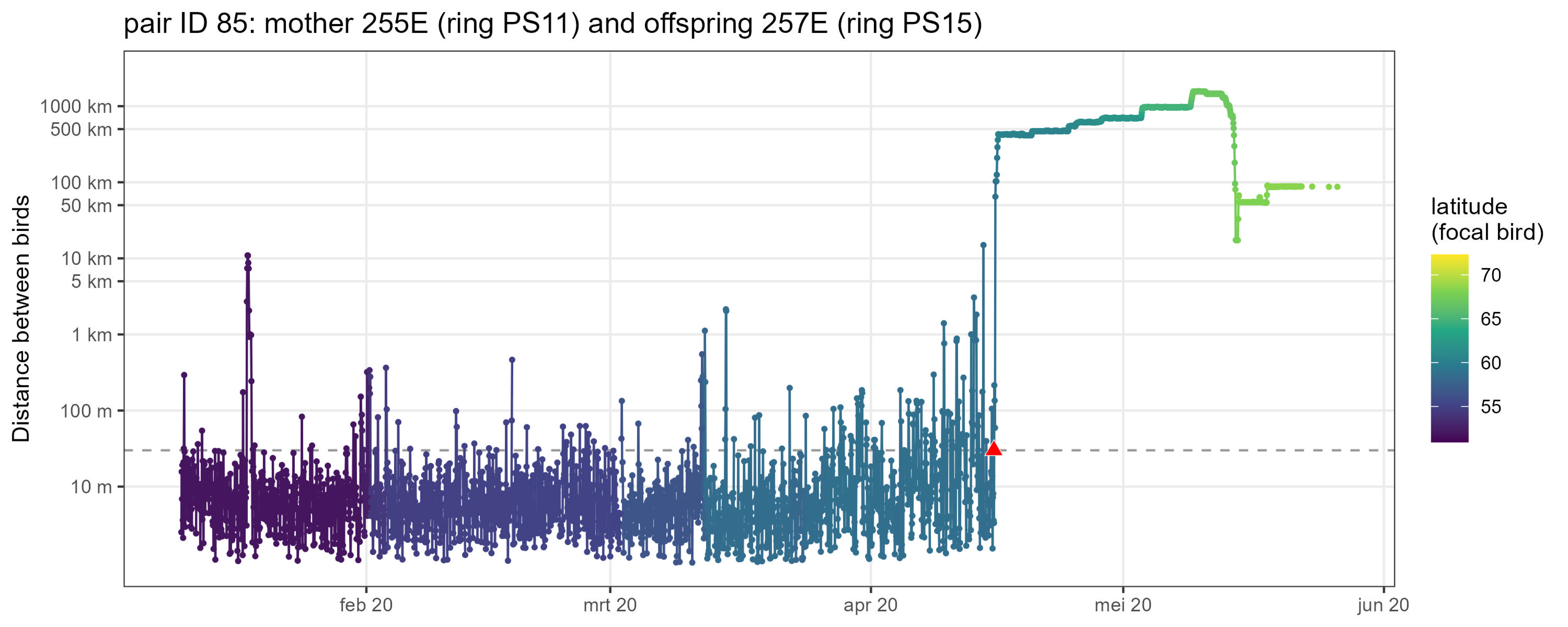
pair ID 85: mother 255E (ring PS11) and offspring 257E (ring PS15)
Distance between birds
1000 km
500 km
100 km
50 km
10 km
5 km
1 km
100 m
10 m
feb 20
mrt 20
apr 20
mei 20
jun 20
latitude
(focal bird)
70
65
60
55

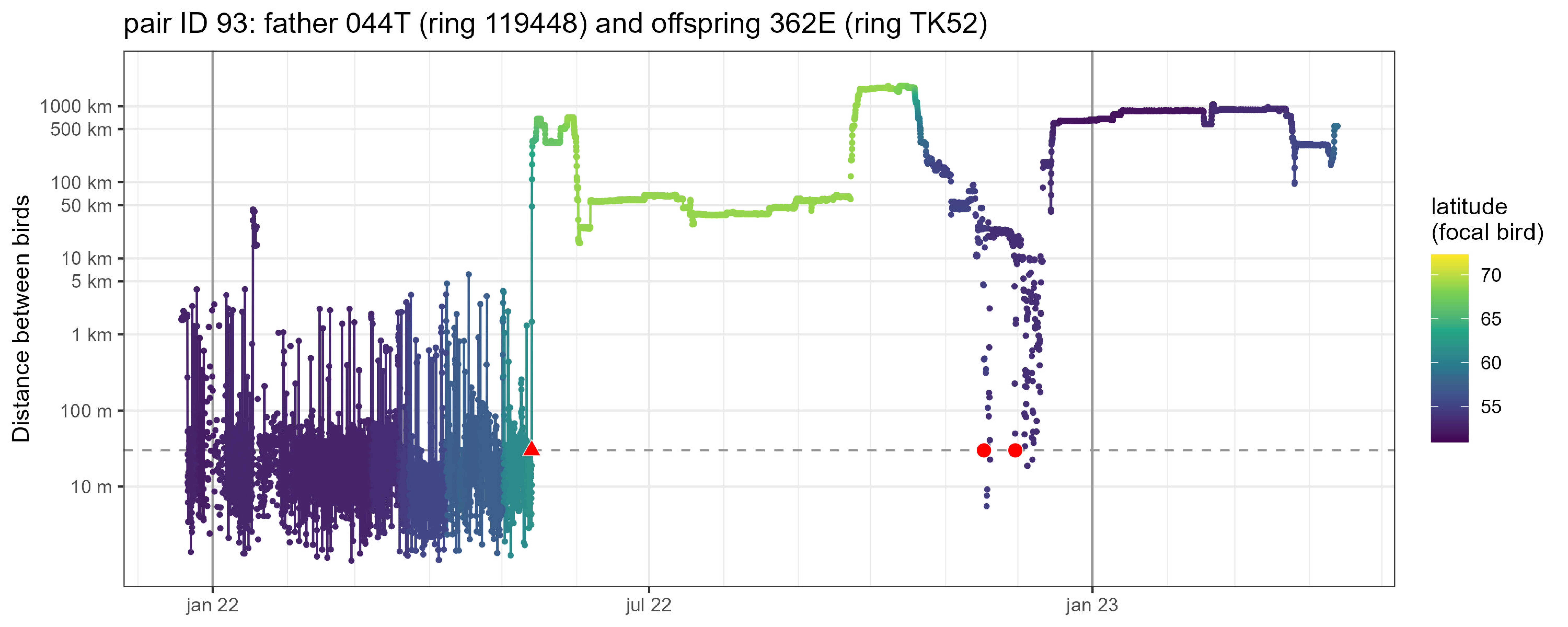
pair ID 93: father 044T (ring 119448) and offspring 362E (ring TK52)
Distance between birds
1000 km
500 km
100 km
50 km
10 km
5 km
1 km
100 m
10 m
jan 22
jul 22
jan 23
latitude
(focal bird)
70
65
60
55

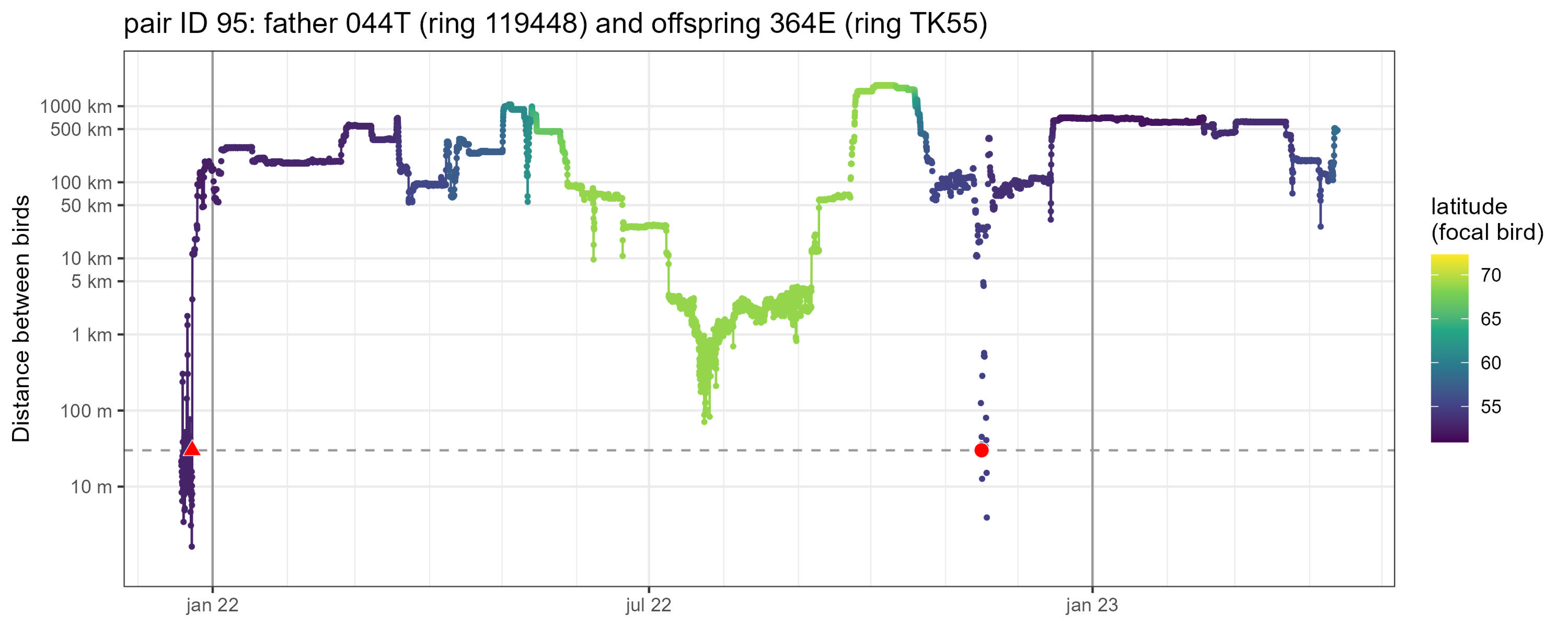

pair ID 95: father 044T (ring 119448) and offspring 364E (ring TK55)
Distance between birds
1000 km
500 km
100 km
50 km
10 km
5 km
1 km
100 m
10 m
jan 22
jul 22
jan 23
latitude
(focal bird)
70
65
60
55

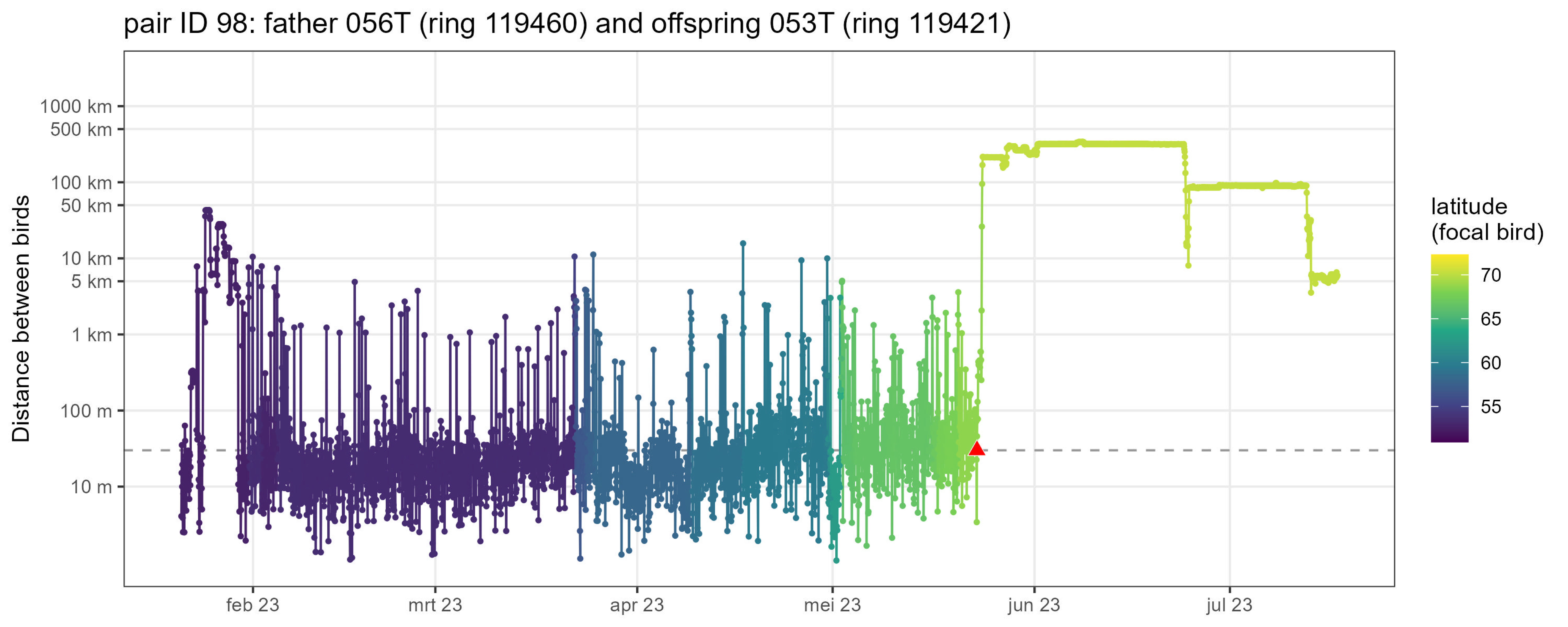
pair ID 98: father 056T (ring 119460) and offspring 053T (ring 119421)
Distance between birds
1000 km
500 km
100 km
50 km
10 km
5 km
1 km
100 m
10 m
feb 23
mrt 23
apr 23
mei 23
jun 23
jul 23
latitude
(focal bird)
70
65
60
55

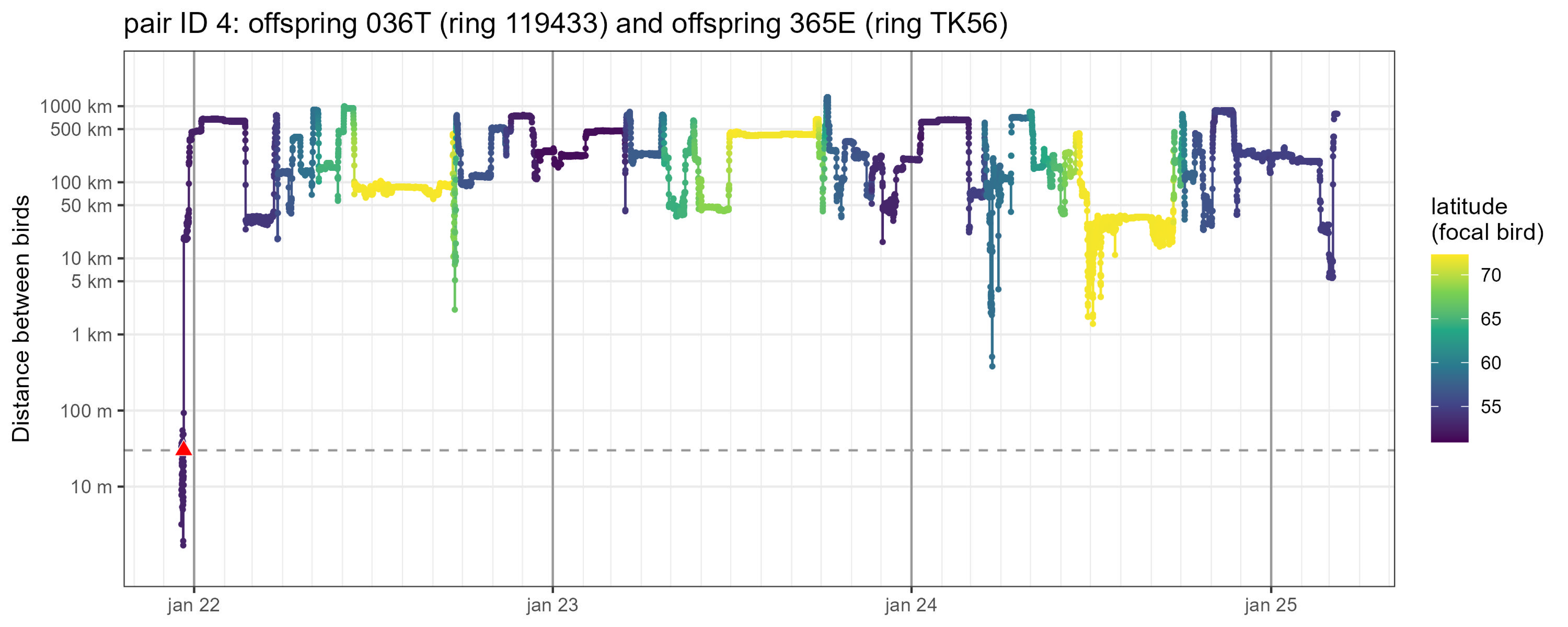
pair ID 4: offspring 036T (ring 119433) and offspring 365E (ring TK56)
Distance between birds
1000 km
500 km
100 km
50 km
10 km
5 km
1 km
100 m
10 m
jan 22
jan 23
jan 24
jan 25
latitude
(focal bird)
70
65
60
55

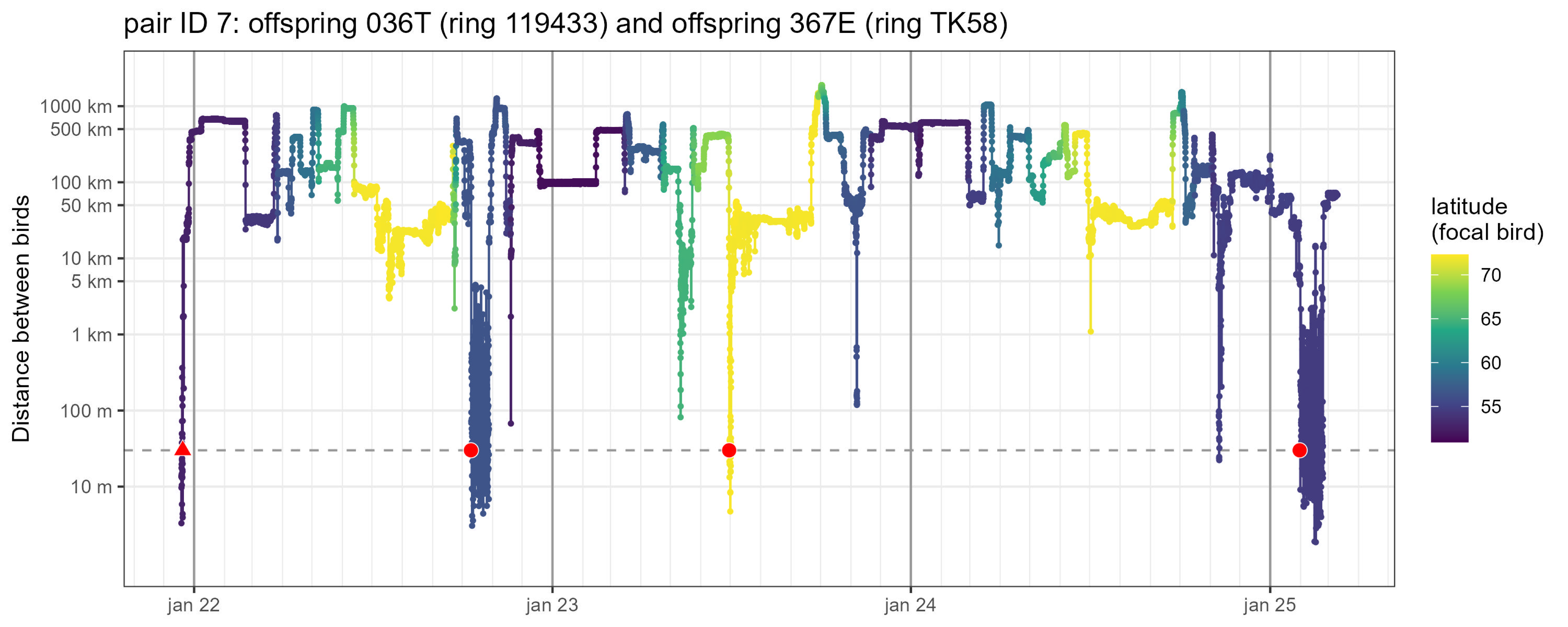

pair ID 7: offspring 036T (ring 119433) and offspring 367E (ring TK58)
Distance between birds
1000 km
500 km
100 km
50 km
10 km
5 km
1 km
100 m
10 m
jan 22
jan 23
jan 24
jan 25
latitude
(focal bird)
70
65
60
55

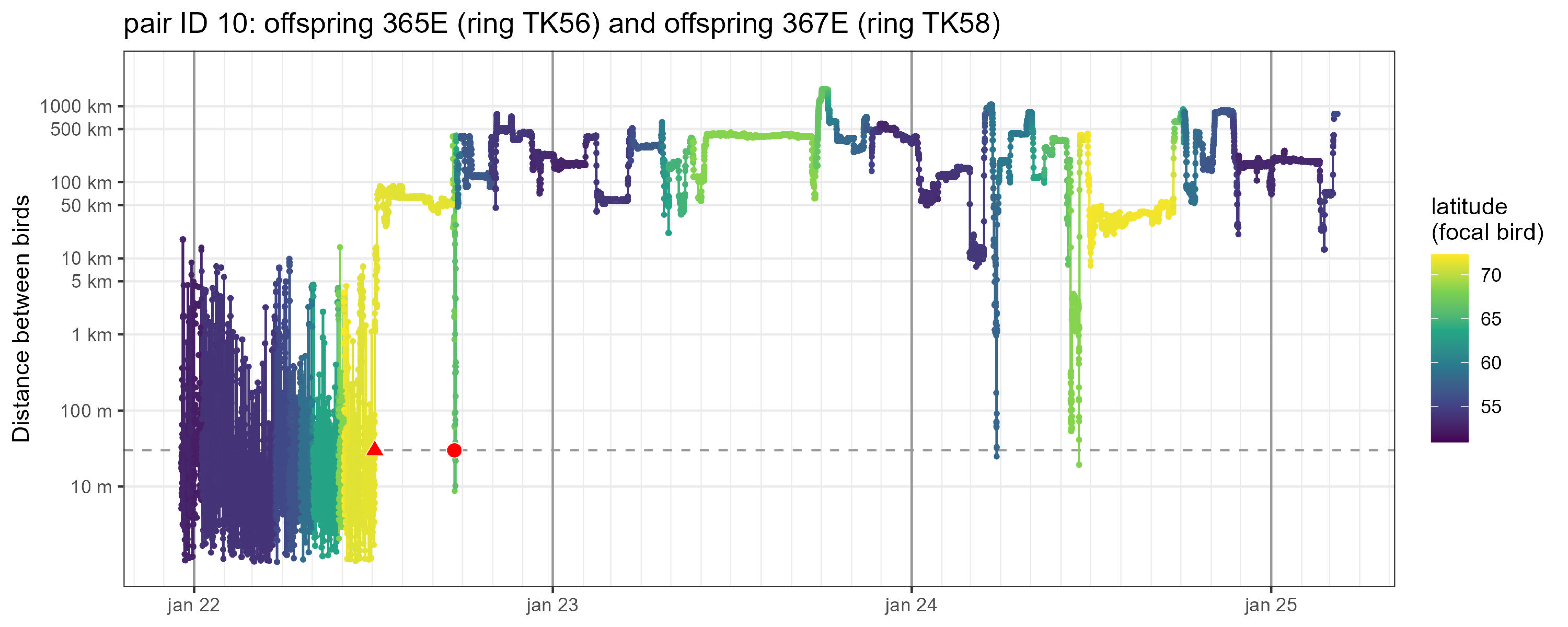
pair ID 10: offspring 365E (ring TK56) and offspring 367E (ring TK58)
Distance between birds
1000 km
500 km
100 km
50 km
10 km
5 km
1 km
100 m
10 m
jan 22
jan 23
jan 24
jan 25
latitude
(focal bird)
70
65
60
55

pair ID 20: offspring 359E (ring 119456) and offspring 360E (ring 119457)

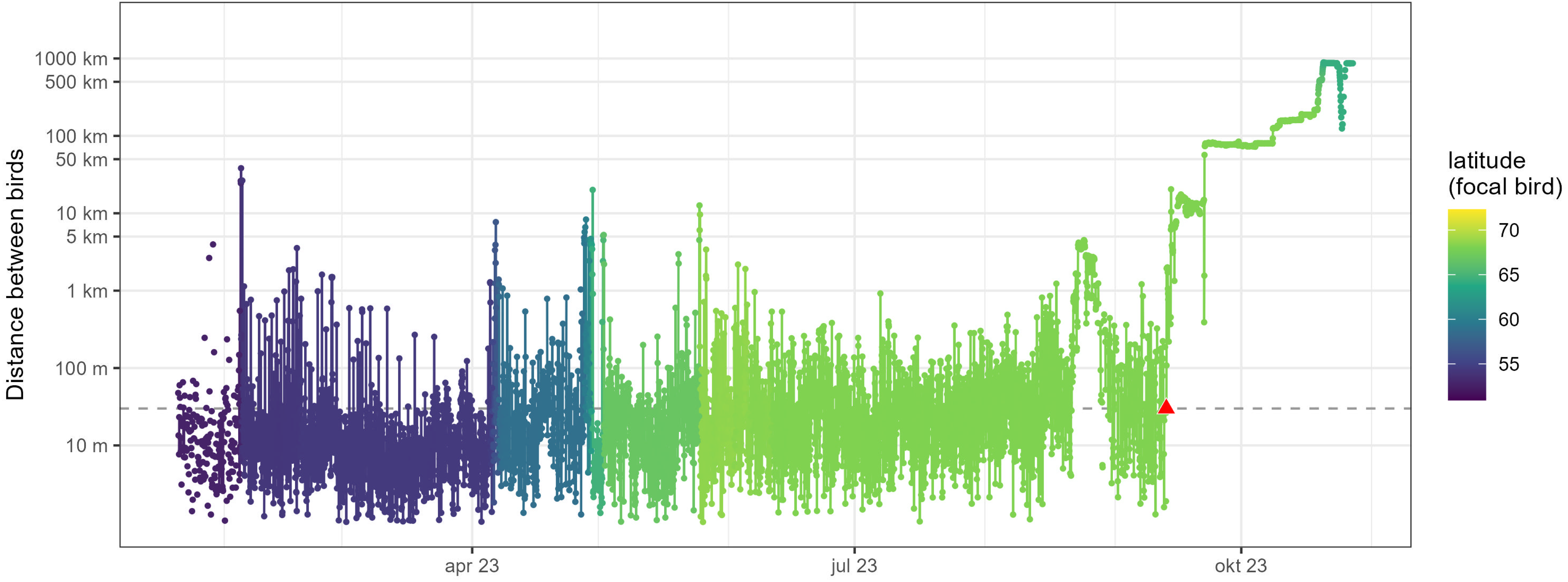

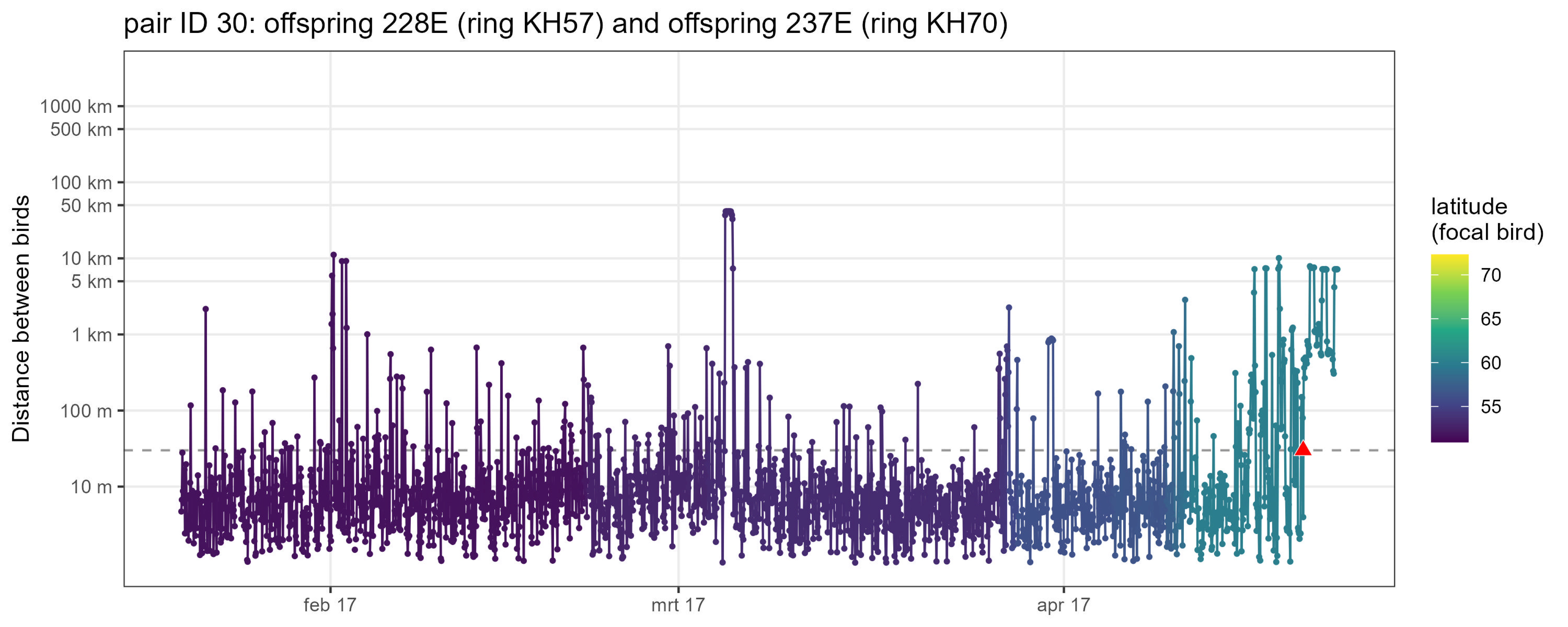

pair ID 30: offspring 228E (ring KH57) and offspring 237E (ring KH70)
Distance between birds
1000 km
500 km
100 km
50 km
10 km
5 km
1 km
100 m
10 m
feb 17
mrt 17
apr 17
latitude
(focal bird)
70
65
60
55

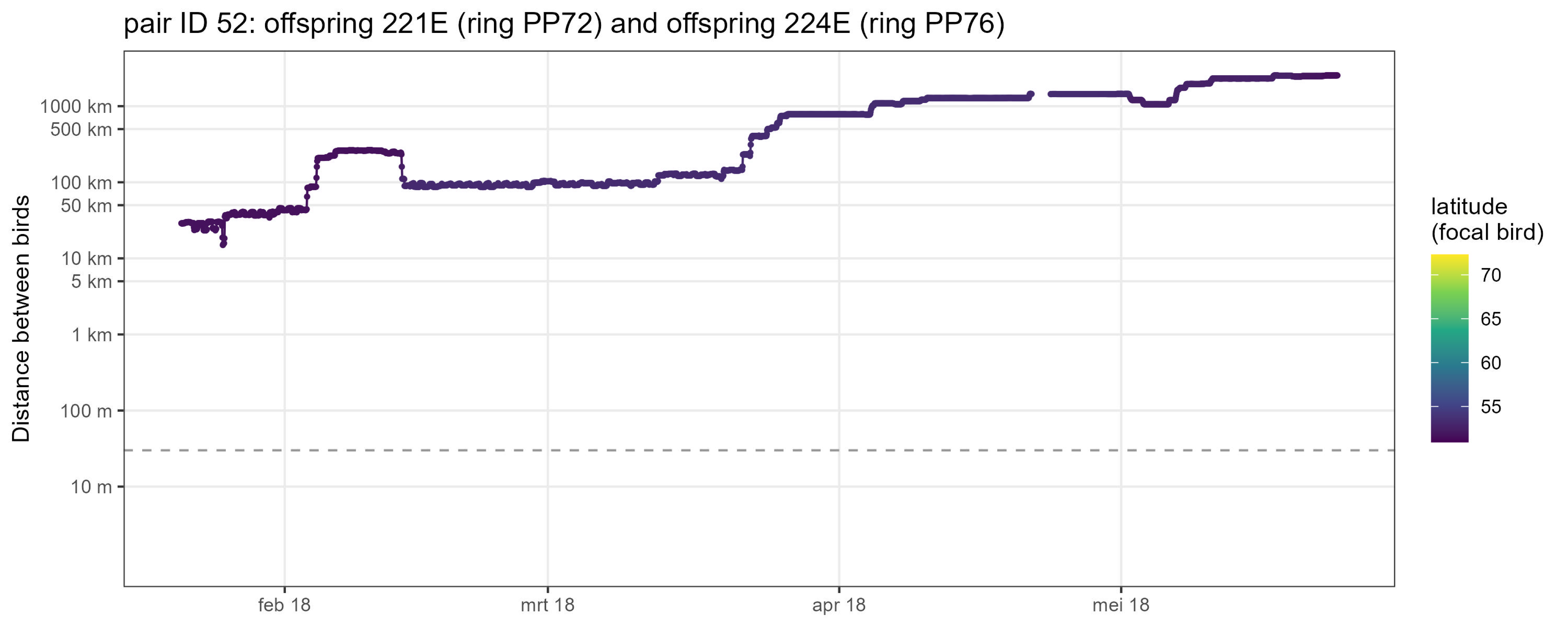

pair ID 52: offspring 221E (ring PP72) and offspring 224E (ring PP76)
Distance between birds
1000 km
500 km
100 km
50 km
10 km
5 km
1 km
100 m
10 m
feb 18
mrt 18
apr 18
mei 18
latitude
(focal bird)
70
65
60
55

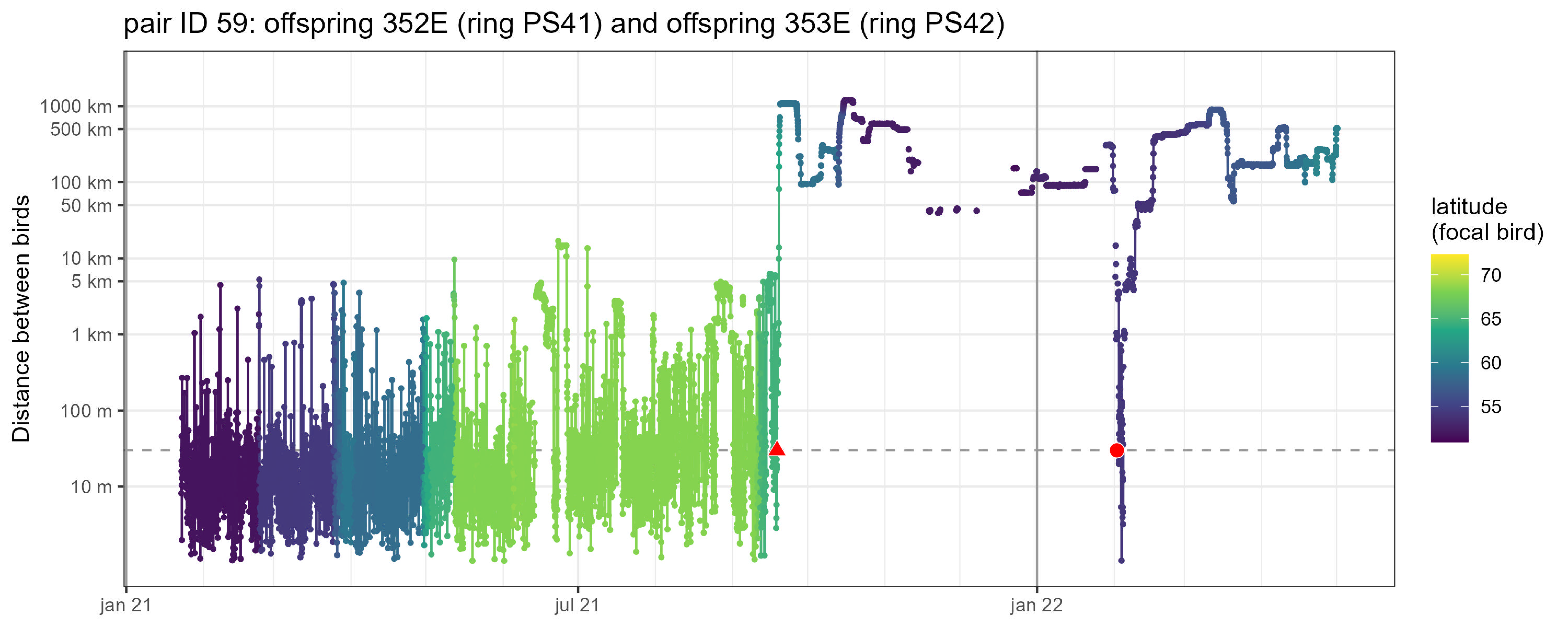

pair ID 59: offspring 352E (ring PS41) and offspring 353E (ring PS42)
Distance between birds
1000 km
500 km
100 km
50 km
10 km
5 km
1 km
100 m
10 m
jan 21
jul 21
jan 22
latitude
(focal bird)
70
65
60
55

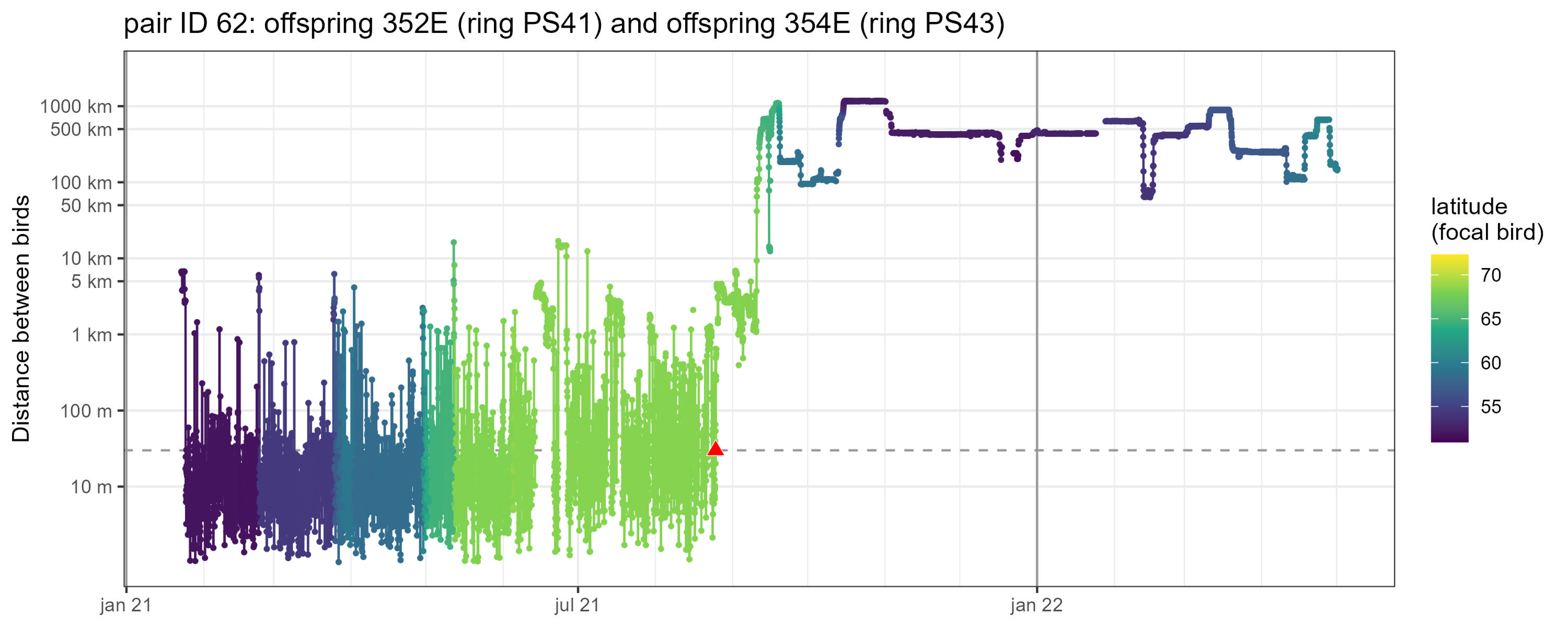
pair ID 62: offspring 352E (ring PS41) and offspring 354E (ring PS43)
Distance between birds
1000 km
500 km
100 km
50 km
10 km
5 km
1 km
100 m
10 m
jan 21
jul 21
jan 22
latitude
(focal bird)
70
65
60
55

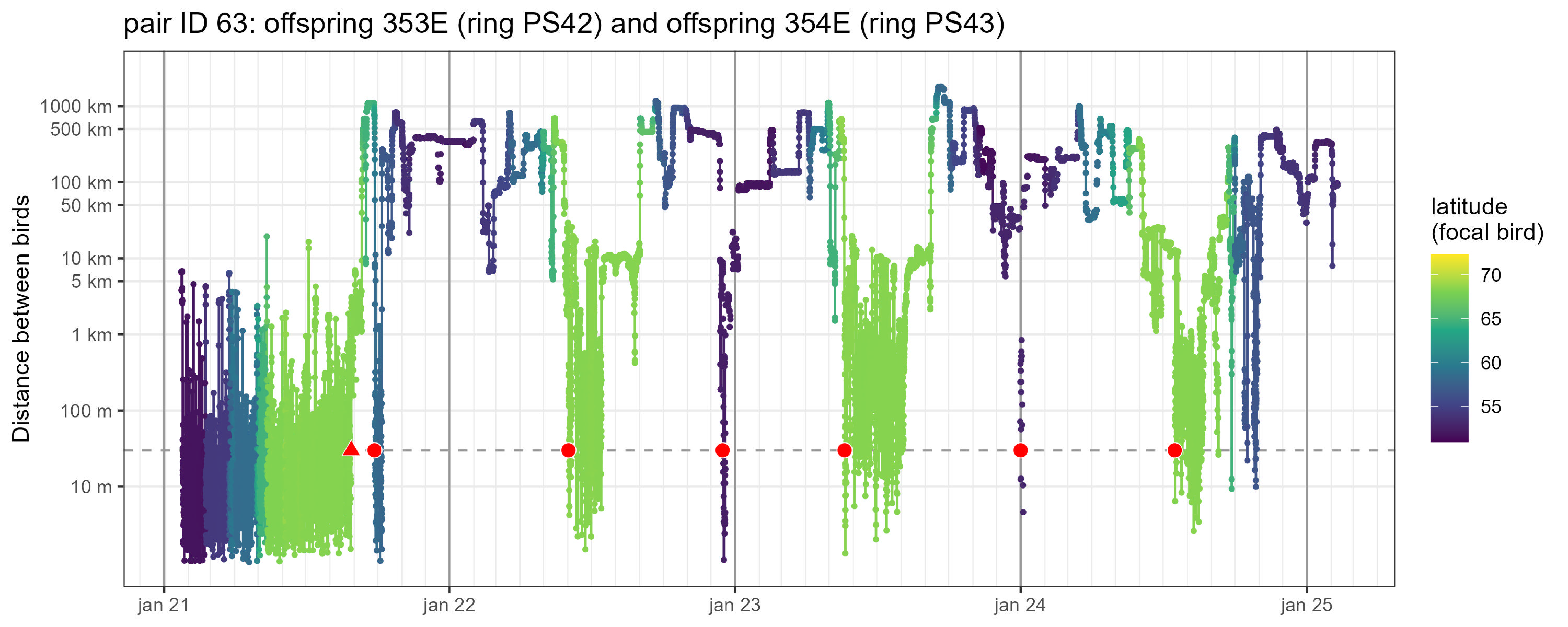

pair ID 63: offspring 353E (ring PS42) and offspring 354E (ring PS43)
Distance between birds
1000 km
500 km
100 km
50 km
10 km
5 km
1 km
100 m
10 m
jan 21
jan 22
jan 23
jan 24
jan 25
latitude
(focal bird)
70
65
60
55

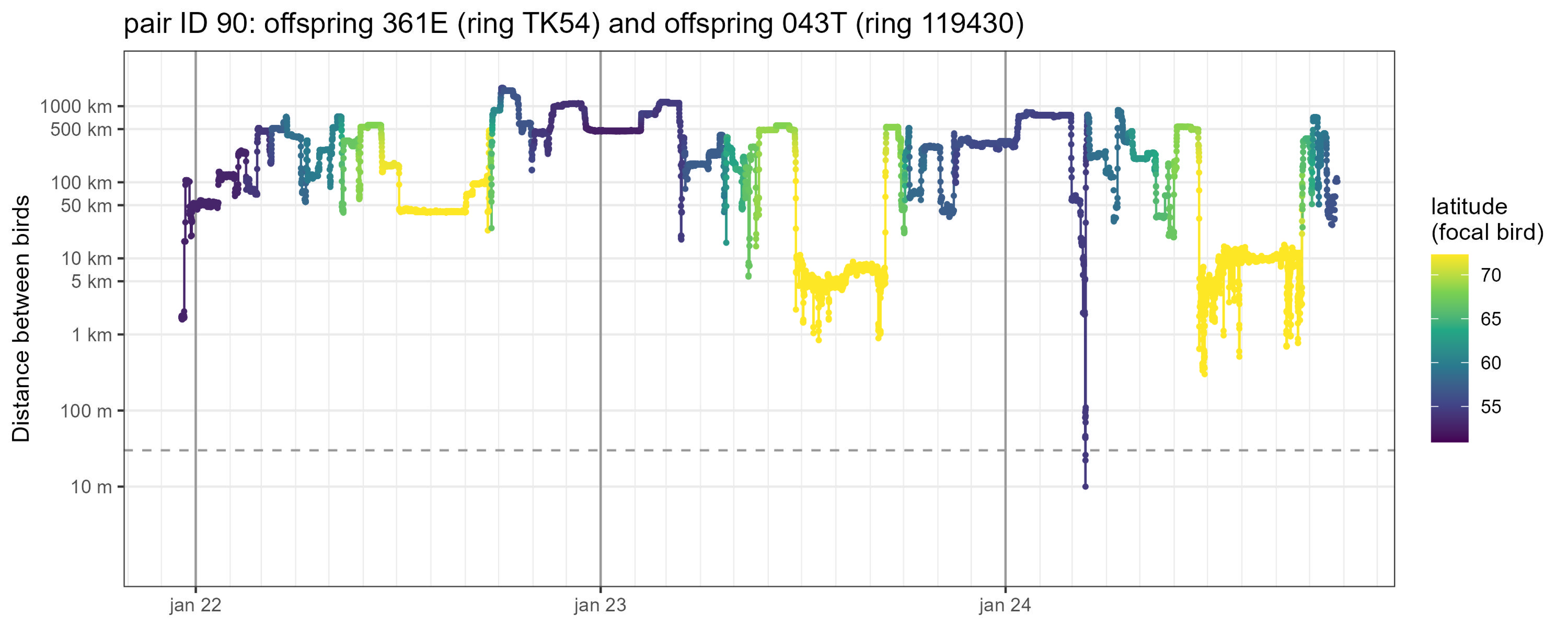
pair ID 90: offspring 361E (ring TK54) and offspring 043T (ring 119430)
Distance between birds
1000 km
500 km
100 km
50 km
10 km
5 km
1 km
100 m
10 m
jan 22
jan 23
jan 24
latitude
(focal bird)
70
65
60
55

pair ID 91: offspring 377E (ring TK72) and offspring 378E (ring TK73)

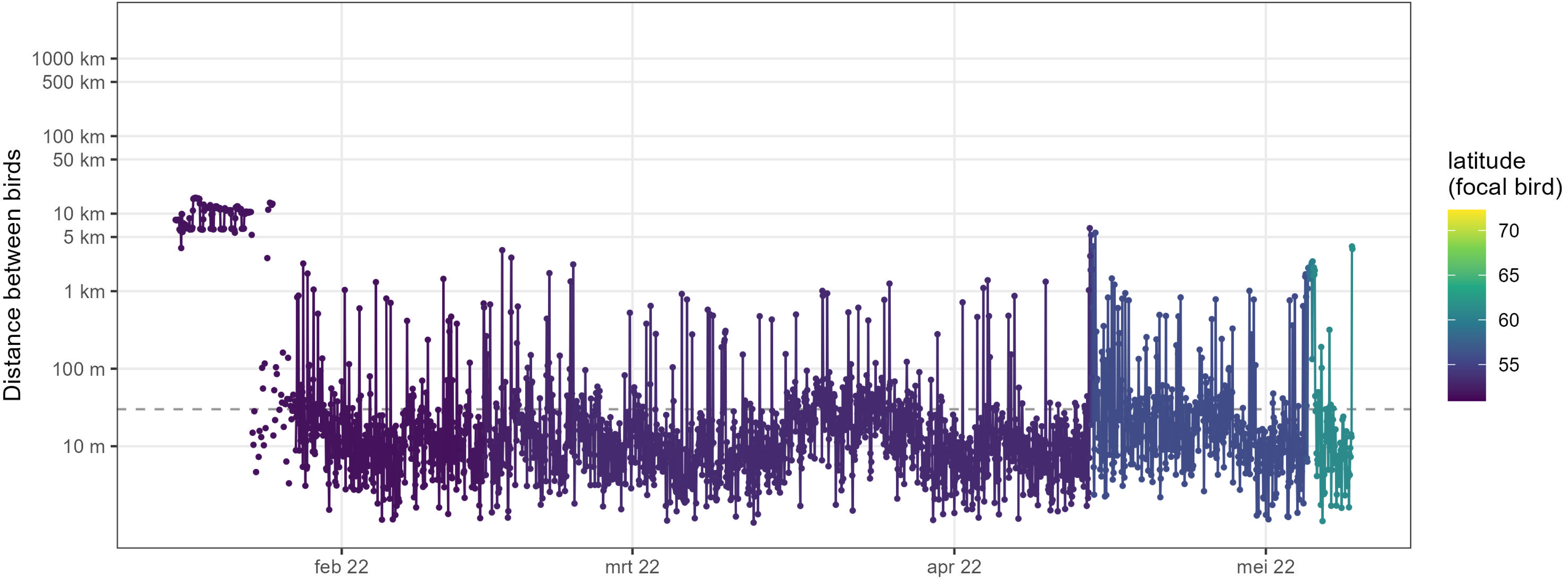

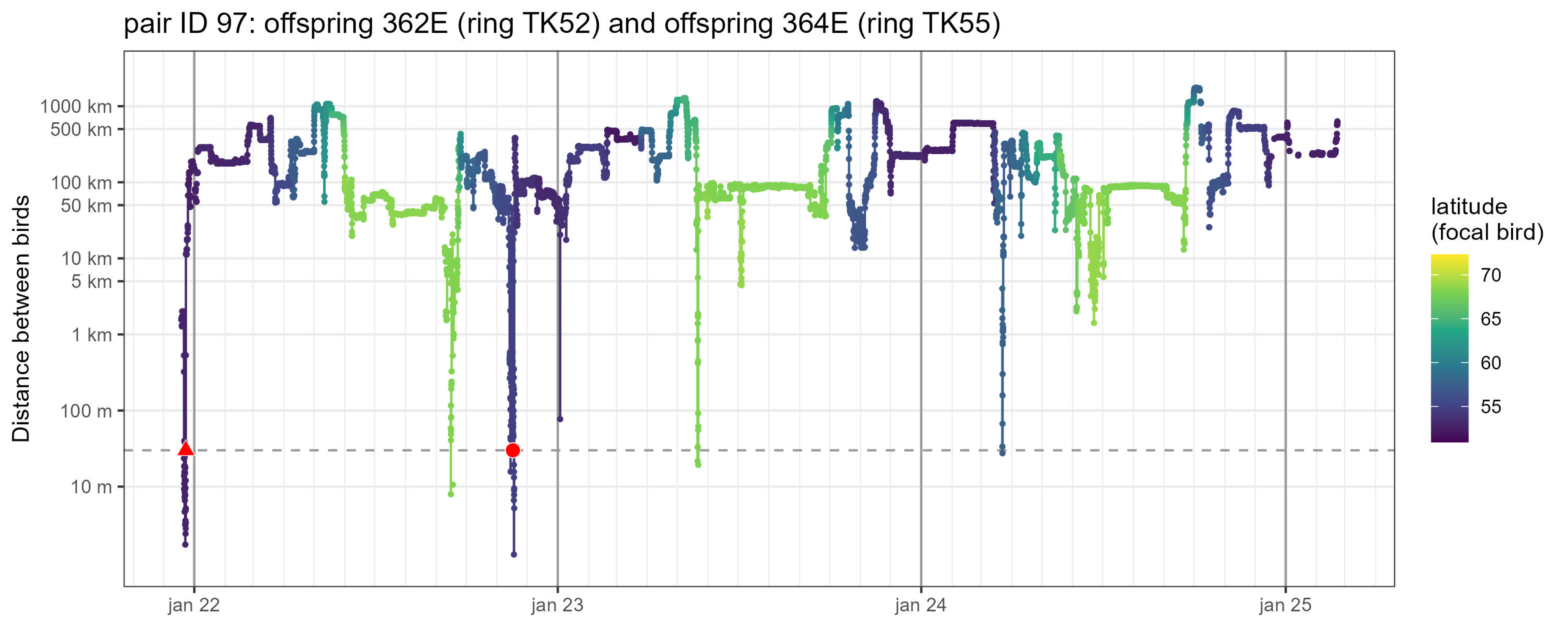
pair ID 97: offspring 362E (ring TK52) and offspring 364E (ring TK55)
Distance between birds
1000 km
500 km
100 km
50 km
10 km
5 km
1 km
100 m
10 m
jan 22
jan 23
jan 24
jan 25
latitude
(focal bird)
70
65
60
55

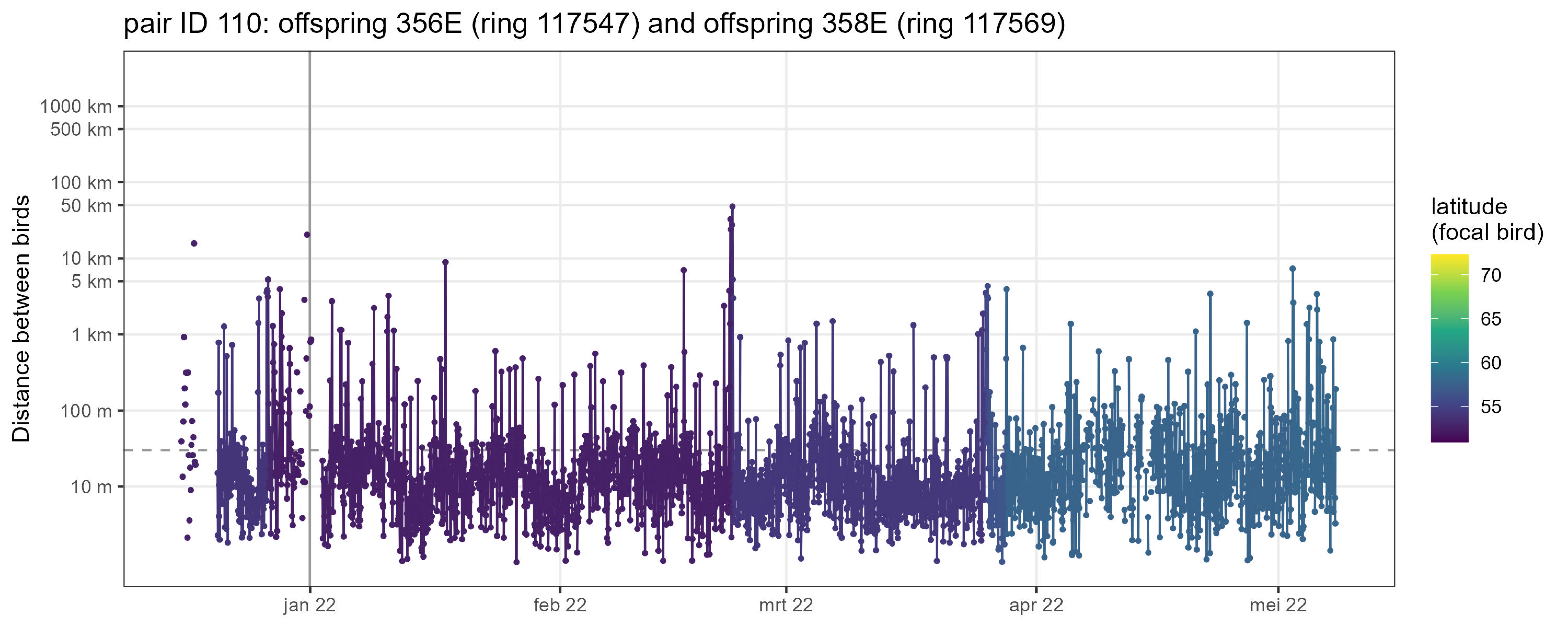

pair ID 110: offspring 356E (ring 117547) and offspring 358E (ring 117569)
Distance between birds
1000 km
500 km
100 km
50 km
10 km
5 km
1 km
100 m
10 m
jan 22
feb 22
mrt 22
apr 22
mei 22
latitude
(focal bird)
70
65
60
55

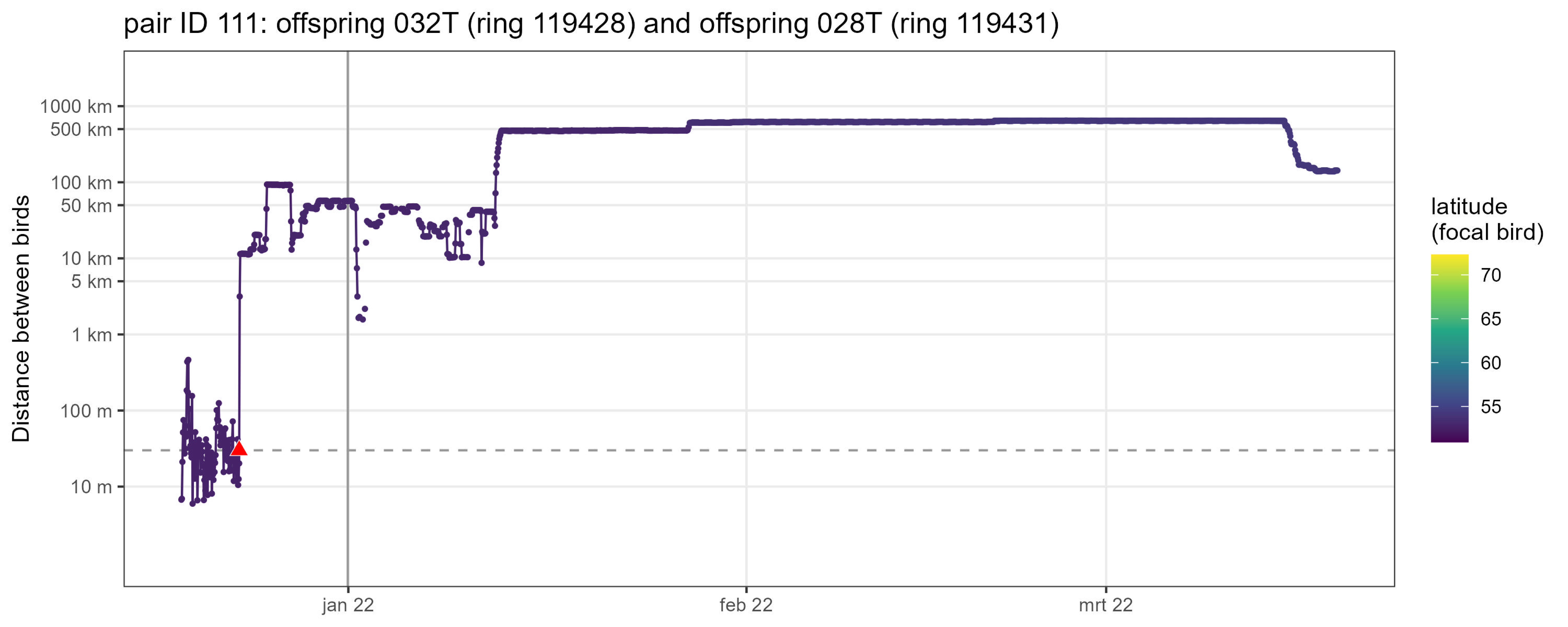

pair ID 111: offspring 032T (ring 119428) and offspring 028T (ring 119431)
Distance between birds
1000 km
500 km
100 km
50 km
10 km
5 km
1 km
100 m
10 m
jan 22
feb 22
mrt 22
latitude
(focal bird)
70
65
60
55

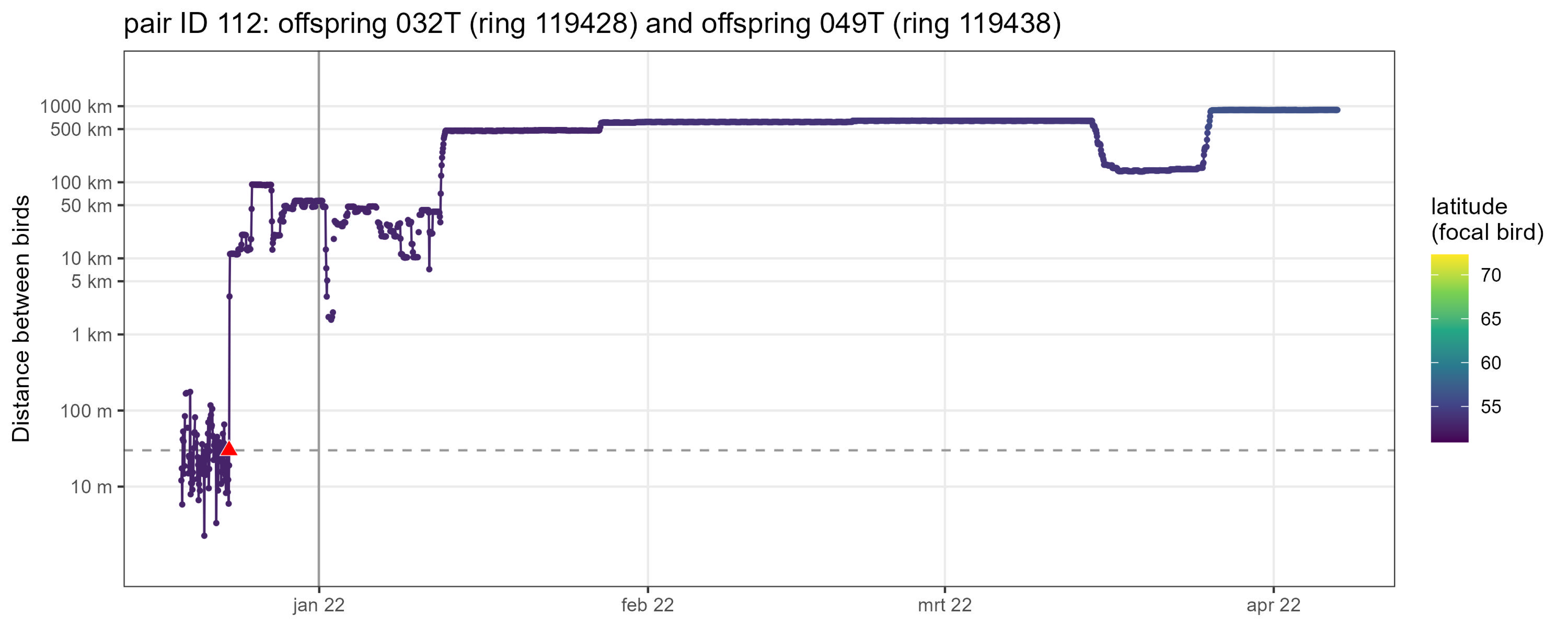
pair ID 112: offspring 032T (ring 119428) and offspring 049T (ring 119438)
Distance between birds
1000 km
500 km
100 km
50 km
10 km
5 km
1 km
100 m
10 m
jan 22
feb 22
mrt 22
apr 22
latitude
(focal bird)
70
65
60
55

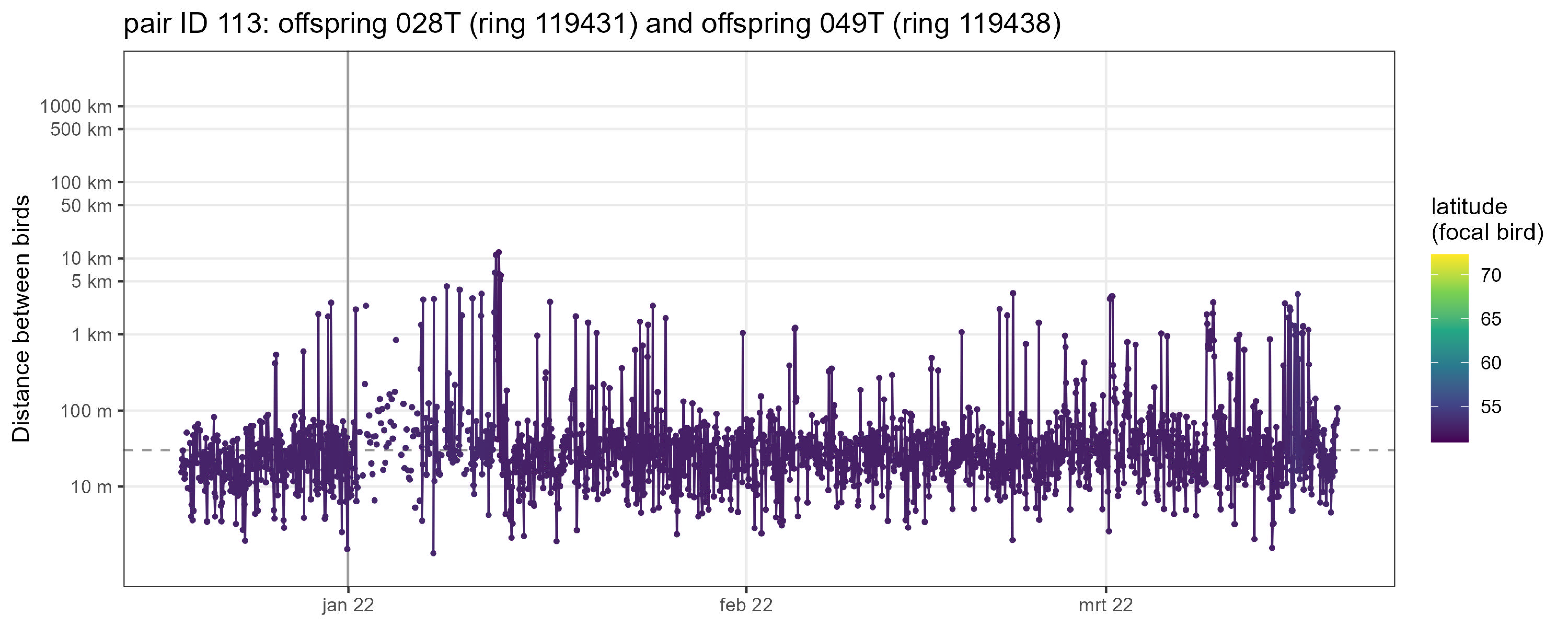
pair ID 113: offspring 028T (ring 119431) and offspring 049T (ring 119438)
Distance between birds
1000 km
500 km
100 km
50 km
10 km
5 km
1 km
100 m
10 m
jan 22
feb 22
mrt 22
latitude
(focal bird)
70
65
60
55

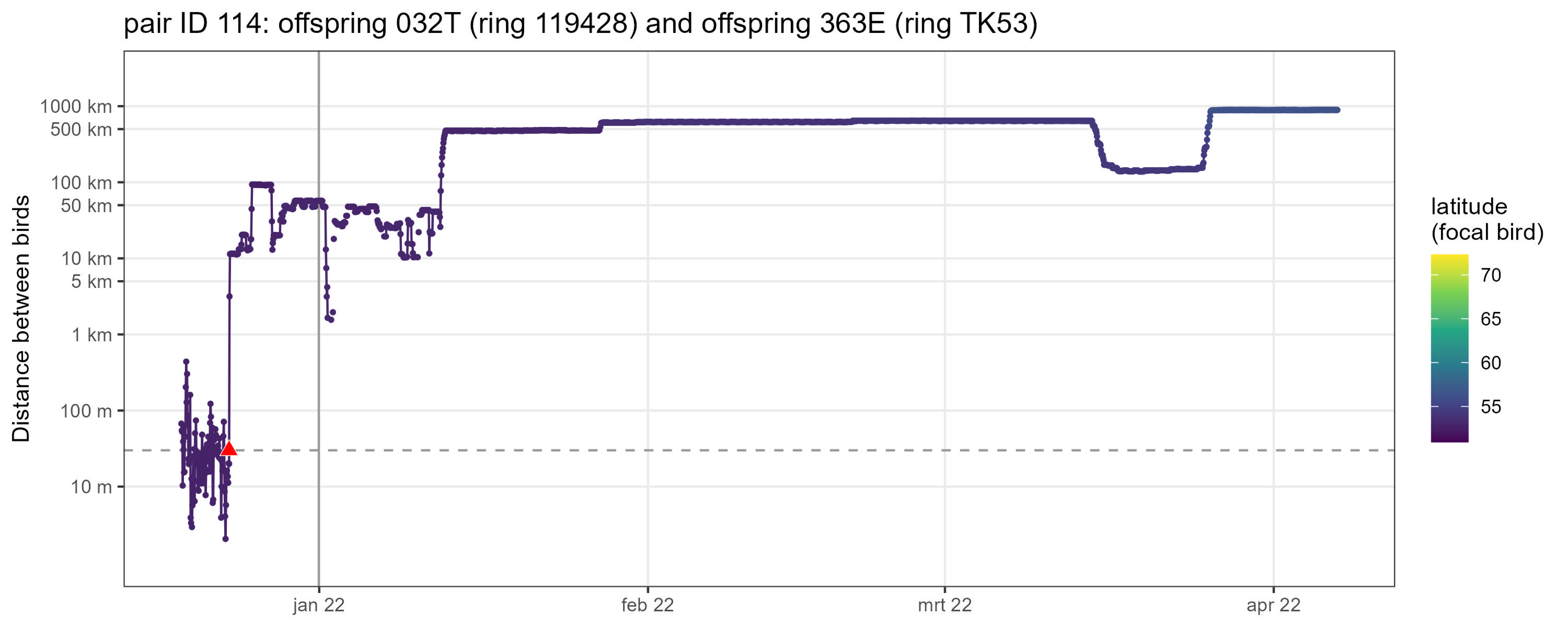
pair ID 114: offspring 032T (ring 119428) and offspring 363E (ring TK53)
Distance between birds
1000 km
500 km
100 km
50 km
10 km
5 km
1 km
100 m
10 m
jan 22
feb 22
mrt 22
apr 22
latitude
(focal bird)
70
65
60
55

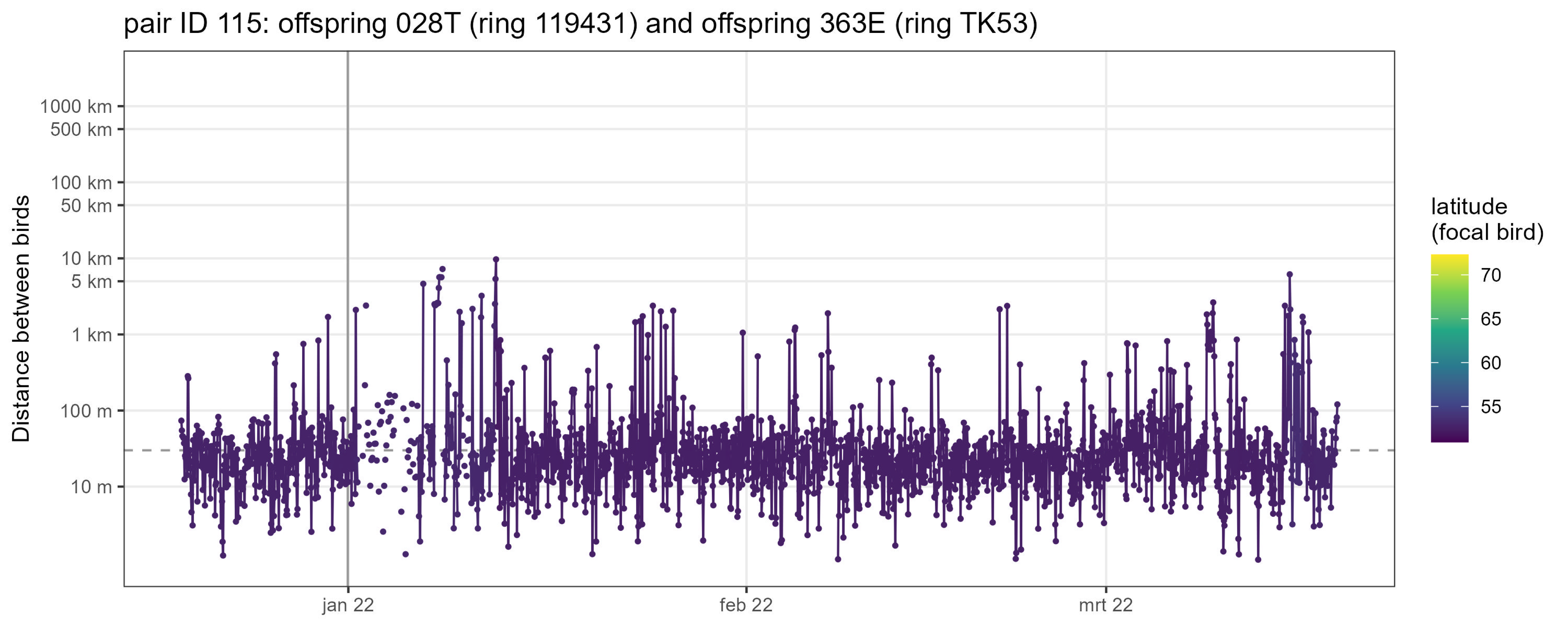
pair ID 115: offspring 028T (ring 119431) and offspring 363E (ring TK53)
Distance between birds
1000 km
500 km
100 km
50 km
10 km
5 km
1 km
100 m
10 m
jan 22
feb 22
mrt 22
latitude
(focal bird)
70
65
60
55

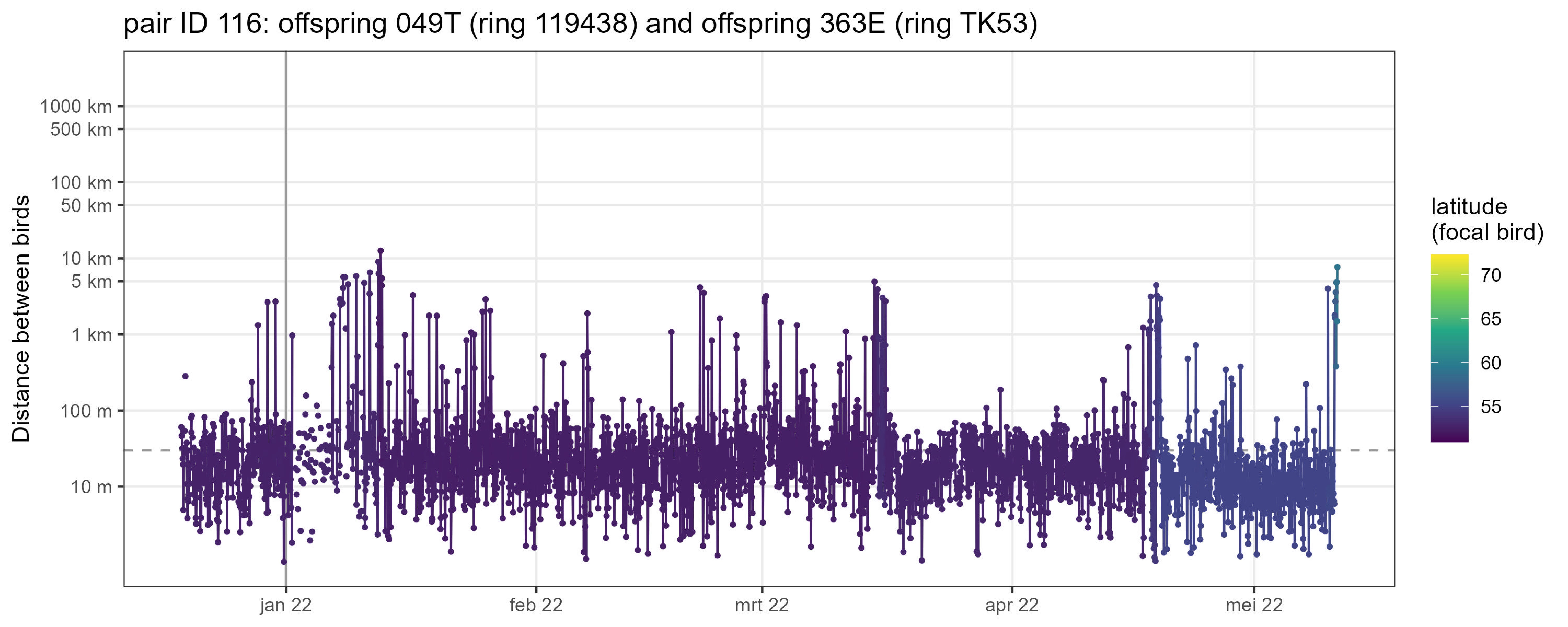
pair ID 116: offspring 049T (ring 119438) and offspring 363E (ring TK53)
Distance between birds
1000 km
500 km
100 km
50 km
10 km
5 km
1 km
100 m
10 m
jan 22
feb 22
mrt 22
apr 22
mei 22
latitude
(focal bird)
70
65
60
55

pair ID 117: offspring 232E (ring KH62) and offspring 218E (ring KH75)

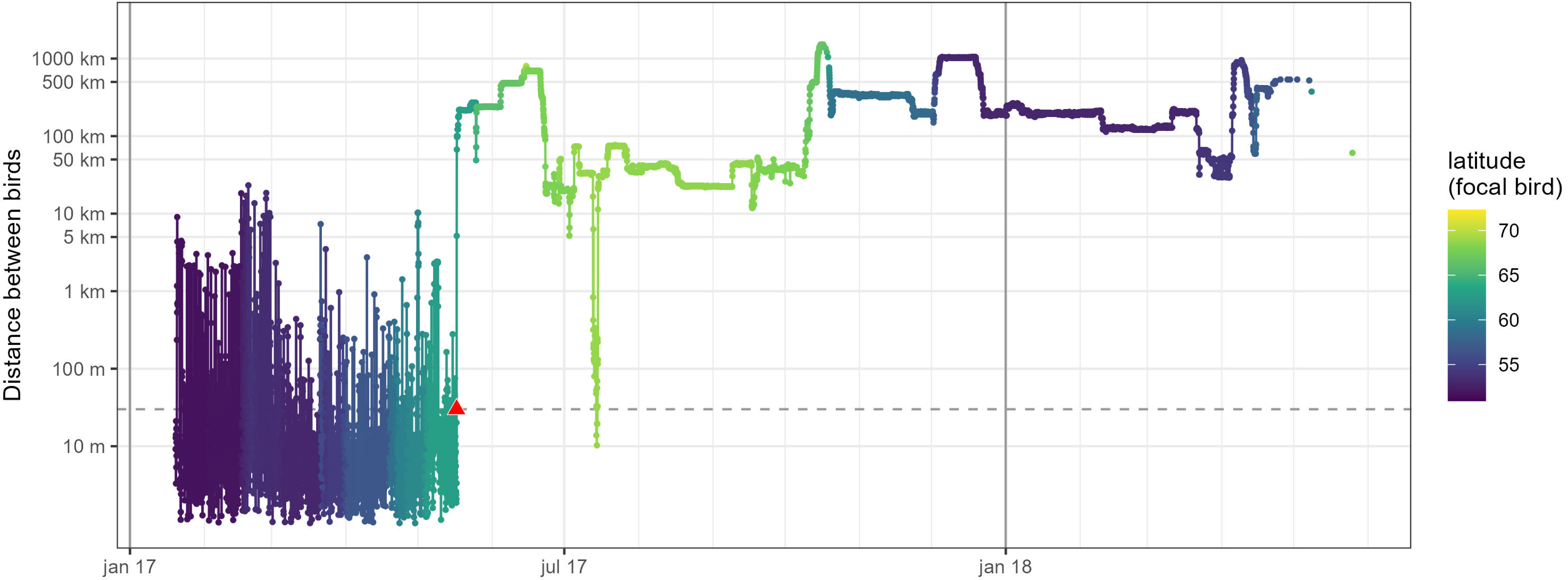

Supplement: Supplementary file 2 [file 265_2026_3787_MOESM2_ESM.pdf]
